# PerseuCPP: a machine learning strategy to predict cell-penetrating peptides and their uptake efficiency

**DOI:** 10.1093/bioadv/vbaf213

**Published:** 2025-09-08

**Authors:** Rayane Monique Bernardes-Loch, Gustavo de Oliveira Almeida, Igor Teixeira Brasiliano, Wagner Meira Jr, Douglas E V Pires, Maria Cristina Baracat-Pereira, Sabrina de Azevedo Silveira

**Affiliations:** Department of Biochemistry and Molecular Biology, Universidade Federal de Viçosa, Viçosa-MG 36570-900, Brazil; Department of Computer Science, Universidade Federal de Viçosa, Viçosa-MG 36570-900, Brazil; Department of Computer Science, Universidade Federal de Viçosa, Viçosa-MG 36570-900, Brazil; Department of Computer Science, Universidade Federal de Minas Gerais, Belo Horizonte-MG 31270-901, Brazil; School of Computing and Information Systems, University of Melbourne, Parkville 3052, Australia; Department of Biochemistry and Molecular Biology, Universidade Federal de Viçosa, Viçosa-MG 36570-900, Brazil; Department of Computer Science, Universidade Federal de Viçosa, Viçosa-MG 36570-900, Brazil; IDATA—Institute of Artificial Intelligence and Computational Science, Universidade Federal de Viçosa, Viçosa-MG 36570-900, Brazil

## Abstract

**Motivation:**

Cell-penetrating peptides (CPPs) are promising tools for transporting therapeutic molecules into cells without damaging the cellular membrane. These peptides serve as efficient drug delivery systems, capable of carrying diverse biologically active substances while exhibiting low cytotoxicity compared to non-native molecules. However, identifying CPPs through experimental methods is expensive and time-consuming, making computational strategies an attractive alternative due to their cost-effectiveness and scalability.

**Results:**

This study introduces PerseuCPP, a machine learning strategy designed to identify CPPs. Based on descriptors including physicochemical and structural properties as well as atomic composition, our strategy employs the Extremely Randomized Trees to predict CPPs and their uptake efficiency. The first stage was developed using a balanced dataset of 967 CPPs and non-CPPs, applying a 10-fold cross-validation scheme. Two independent datasets were utilized for validation. The CPP predictor achieved superior results compared to state-of-the-art methods, with MCC 0.854, Recall 0.860, and AUC 0.984. The second stage, focused on efficiency prediction, was trained on a balanced dataset of 140 CPPs and non-CPPs, also using a 10-fold cross-validation scheme, and validated with an independent dataset. The efficiency predictor achieved competitive results, with Recall 0.761 and AUC 0.690. PerseuCPP is interpretable, offering insights into the key descriptors enabling peptides to penetrate cells effectively. We anticipate that PerseuCPP will be a valuable tool for advancing the design and application of CPPs in drug delivery and biomedical research.

**Availability and implementation:**

https://github.com/goalmeida05/PERSEU.

## 1 Introduction

One of the most remarkable achievements of medicine in the 20th century was the discovery of antibiotics (Gaynes 2017, Nature Editorial 2018). Their use in clinical routine for treating bacterial infections significantly reduced patient mortality. During the so-called “Golden Age of antibiotics,” there were significant discoveries of new antibacterial drugs (Gaynes 2017).

However, despite the existence of these drugs, bacterial resistance began to be observed. The Center for Disease Control (CDC) estimates that nearly 2 million deaths occur in the United States due to bacterial resistance to first-line antibiotics (CDC 2019). Almost all known bacterial species have some gene that leads to antibiotic resistance (Li *et al.* 2023). Bacterial species resistant to antibiotics were also detected in several environmental sites, such as surface water bodies, agricultural soils, and animal manure (Liu *et al.* 2023). Given this scenario, it is imperative to search for new molecules with potential to circumvent antibiotic resistance.

Cell-penetrating peptides (CPPs) are strong contenders in addressing this problem. CPPs are characterized by a short chain of amino acids (usually up to 30 residues in length), partially hydrophobic, usually with a net positive charge, as they are rich in arginine and lysine and show a high isoelectric point (Ramsey and Flynn 2015). CPPs are known for their ability to transport various therapeutic molecules into cells, including antibiotics (Hadjicharalambous *et al.* 2022, Kravchenko *et al.* 2023, Mullard 2023). This can potentially enhance the treatment efficacy and overcome some resistance mechanisms (Ramsey and Flynn 2015). An example is the peptide transactivator of transcription from HIV (TAT), which is well-known for its ability to transport therapeutic molecules into cells (Gulseren *et al.* 2015). Additionally, using CPPs to transport antibiotics directly into resistant bacteria can reduce the necessary antibiotic concentration, minimizing side effects and decreasing the development of additional resistance (Ramsey and Flynn 2015).

In structural bioinformatics, natural residues are the 20 proteinogenic canonical amino acids encoded by the genetic code (alanine, glycine, lysine, etc.), which form the basis of proteins and peptides found in nature (Alberts *et al.* 2002, Voet *et al.* 2011). In contrast, non-natural amino acids (or non-canonical residues) include synthetic analogues, D-amino acids, and chemically modified derivatives incorporated into peptides to enhance stability or binding affinity; however, these residues are rarely well represented in experimental CPP databases (Kumar *et al.* 2018). Therefore, due to data availability, compatibility with analysis tools, and biological relevance in membrane–peptide interactions, most CPP prediction methods focus exclusively on sequences composed of natural residues.

Determining a peptide’s ability to penetrate cells through *in vitro* and *in vivo* experimental methods is expensive and time-consuming. Therefore, computational methods such as machine learning (ML) (Daina and Zoete 2016, Rodríguez-Pérez *et al.* 2018, Galúcio *et al.* 2019), molecular modeling tools (Pires *et al.* 2015, Blanco *et al.* 2018, Kong *et al.* 2020, Dai *et al.* 2021) and probabilistic models (Dimitri and Lió 2017, Stokes *et al.* 2020) have been applied to identify the most crucial characteristics for analysis. The goal is to facilitate and enhance the accuracy of predictions, allowing for a more streamlined approach without the need for extensive experimental assays.

In an effort to improve the classification of CPPs, recent studies (Gautam *et al.* 2013, Holton *et al.* 2013, Wei *et al.* 2017, de Oliveira *et al.* 2021, Manavalan and Patra 2022, Zhang *et al.* 2023) have investigated the integration of various descriptors from sequence, structure, and physicochemical properties. In Supplementary Section 1, available as supplementary data at *Bioinformatics Advances* online, we briefly review some representative examples of the mentioned studies.

Despite significant progress of predictive methods for CPPs, some limitations persist. High computational cost is a significant issue, particularly for models like the k-skip-n-gram, which, while effective in capturing spatial information, can become inefficient and resource-intensive when applied to large datasets. Moreover, deep learning approaches require substantial computational resources and large training datasets, which might not be accessible to all researchers. These models also tend to lose interpretability, making it challenging to understand the underlying factors contributing to their predictions.

To overcome these challenges, we propose PerseuCPP, an interpretable strategy that leverages descriptors related to structural, physicochemical properties, and atomic composition. We aim to address key limitations present in previous CPP prediction models, more specifically, lack of interpretability, limited generalizability across datasets, and dependence on deep learning architectures with high computational costs. We propose a novel method that models each natural peptide as a feature vector containing the aforementioned descriptors. Next, the resulting matrix is fed into an Extremely Randomized Trees (ERT) algorithm to assess whether a given peptide possesses a cell-penetrating function. A similar process was conducted to train the efficiency classifier. Our results demonstrate the superior predictive capability of PerseuCPP and highlight the biological significance of the selected descriptors for CPP prediction.

Beyond predictive performance, an important contribution of PerseuCPP lies in its interpretability. By relying on biologically meaningful descriptors (e.g. physicochemical, structural, and atomic properties), PerseuCPP enables direct inspection of which features are driving a given prediction. This allows us to: (i) formulate hypotheses on the role of specific properties such as charge distribution or amino acid composition in CPP activity; (ii) investigate why certain sequences are misclassified and whether they may in fact represent yet-undiscovered CPPs; and (iii) guide rational peptide design by suggesting which properties could be modified to enhance CPP. Such insights are not readily available from black-box embeddings alone, where the underlying decision boundaries are not biologically interpretable. In this way, PerseuCPP not only achieves high-quality predictive performance but also provides a framework to connect computational predictions with experimental peptide discovery and design, thereby highlighting its impact beyond classification accuracy.

## 2 Methods

**Figure 1.**
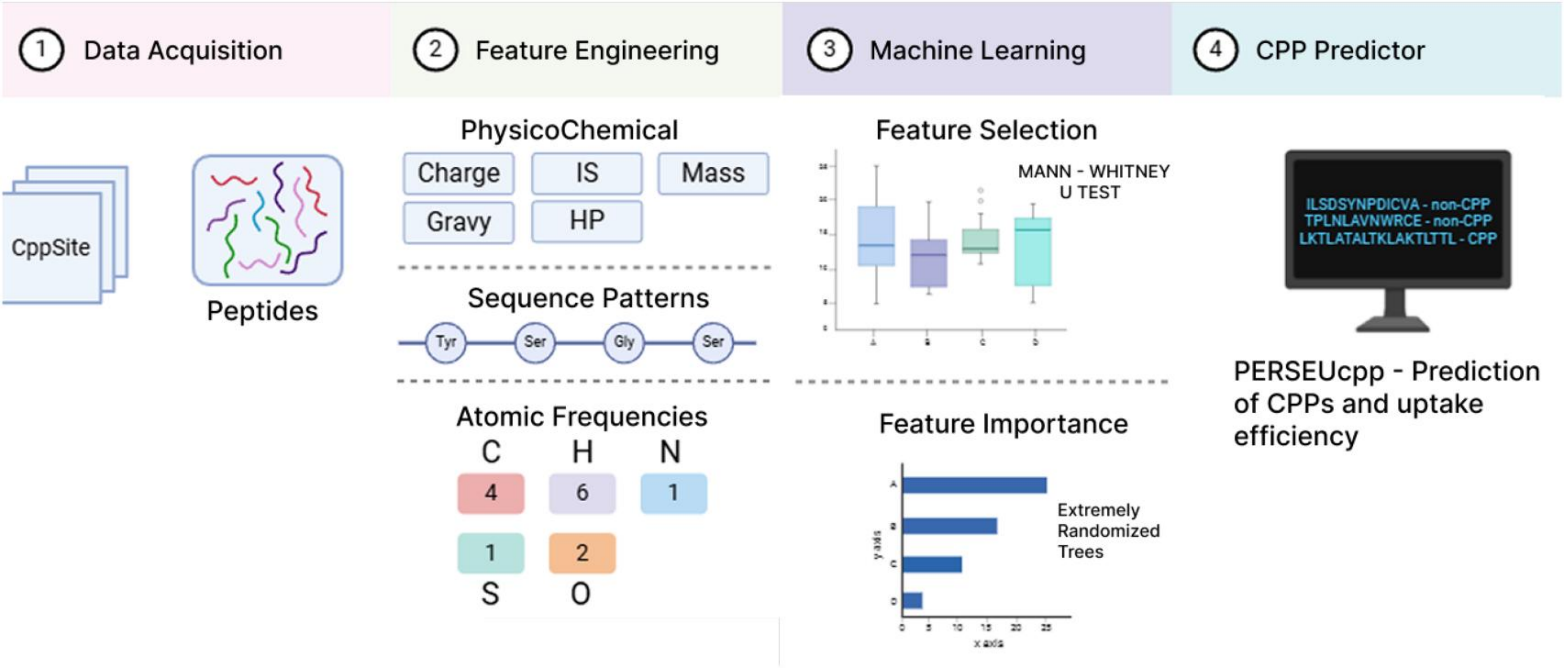
PerseuCPP workflow. The workflow is segmented into four blocks. 1 Data acquisition, peptides sequences was acquired from CppSite2.0 and MLCPP2.0; 2 feature engineering, which involved calculating three classes of descriptors: (i) physicochemical properties, (ii) sequence patterns, which calculate frequencies of residues in the peptide’s chain; (iii) atomic frequencies, which calculate frequencies of each atom in peptide’s chain; 3 were then utilized for training and testing models via supervised learning, with feature selection conducted for model optimization. 4 Best-performing model was implemented, and all the source code is freely available at GitHub.

This section details PerseuCPP, our supervised learning strategy based on structural, physicochemical and atomic composition descriptors to predict CPPs. Here we explain how data were collected and prepared, the encode of CPPs as feature vectors, the supervised learning step as well as the evaluation strategy. Figure 1 presents a workflow that summarizes the main steps of our method.

### 2.1 Dataset preparation

Two datasets were used to train our method, one for CPP classification and another for CPP uptake efficiency classification. Three independent datasets were used in the evaluation to demonstrate its generalization capabilities and to compare PerseuCPP with alternative methods. In the literature, there are datasets that are a composition of existing ones. Therefore, below, we detail all the datasets that were used in this work, directly or indirectly.

1. **CPPSite2.0:** Composed of 1883 unique CPPs (Kardani and Bolhassani 2021). CPPsite2.0 is a continuously updated dataset of experimentally validated CPPs. It was used to augment our training dataset, which we named *PerseuCPP training dataset*.
2. **MLCPP2.0 training dataset:** Originally composed of 573 experimentally validated CPPs from CPPSite2.0 and 573 non-CPPs. The non-CPPs share less than 15% sequence identity with non-CPPs and with CPPs (Manavalan and Patra 2022) in this set.
3. **PerseuCPP training dataset:** Our dataset is composed of the MLCPP2.0 training set, augmented with additional positive sequences from CPPSite2.0. To balance the number of newly added positive sequences with negatives, we downloaded data from several previously published methods (Gautam *et al.* 2013, Holton *et al.* 2013, Wei *et al.* 2017, de Oliveira *et al.* 2021, Manavalan and Patra 2022, Zhang *et al.* 2023), then excluded any sequences that appeared in the test sets (MLCPP2.0 independent dataset, CPP924 independent dataset, CPP1708 test dataset) and removed duplicates. The resulting dataset contained natural peptides with 967 CPPs and 967 non-CPPs, which we used to build our classification model. It is important to note that the negative sequences used in our study—as well as those employed by other models—are not all experimentally validated. In many cases, authors artificially generate negative examples based on sequences that have been experimentally confirmed as non-CPPs, often assuming that peptides with structural or functional similarity to known non-CPPs are likewise non-CPPs.
4. **MLCPP2.0 efficiency training dataset:** Composed of 140 peptides with low and 140 CPPs with high uptake efficiency. This dataset was used to build our model for CPP uptake efficiency classification task, and we named it *train-dataset-2*.
5. **MLCPP2.0 independent dataset:** Composed of 157 experimentally validated CPPs and 2170 non-CPPs. These data were used to compare PerseuCPP with competitors.
6. **MLCPP2.0 efficiency independent dataset:** Composed of 40 CPPs with low and 40 CPPs with high uptake efficiency. The purpose of this dataset was to compare PerseuCPP with competitors (Manavalan and Patra 2022).
7. **CPP924 independent dataset:** A high-quality dataset consisting of 462 unique natural CPPs experimentally validated and non-CPPs. The sequence similarity between any two sequences in the positive group was less than 20% (Wei *et al.* 2017).The purpose of this dataset was to compare PerseuCPP with competitors.
8. **CPP1708 dataset:** Composed of 854 experimentally validated CPPs and 854 non-CPPs (Imre *et al.* 2025). The dataset was generated by deduplicating sequences from multiple sources using CD-HIT at a 70% similarity threshold and balancing the number of positive and negative samples through random sampling. Stratified sampling based on molecular weight was applied to create an 80/10/10 split for training, validation, and testing.

### 2.2 Feature engineering

In the first part of this study, a set of 8831 descriptors was calculated, normalized by the chain length of each peptide sequence. These descriptors included Amino Acid Composition (AAC), Atomic Composition, DiPeptide Composition (DPC), TriPeptide Composition (TPC), Composition of k-spaced Amino Acid Group Pairs (CKSAAGP), Physicochemical properties such as Grand Average of Hydropathy (GRAVY) index, Molecular Mass, Isoelectric Point, Hydrophobicity, Net Charge.

We employed the AAC, a straightforward yet widely utilized feature descriptor of considerable importance for peptide composition. This descriptor computes the frequency of each of the 20 amino acids occurring in peptide sequences and their respective normalization within each chain.

For DPC, all possible combinations of the 20 amino acids, taken pairwise, were computed. Also, we used DPC normalized by the peptide length. For TPC, all possible combinations of the 20 amino acids taken in groups of three were calculated. Additionally, we used TPC normalized by the length of the peptide.

For the CKSAAGP, we calculated the frequency of pairs of amino acid groups separated by *K* residues in the peptide sequence. Amino acids were grouped based on their physicochemical properties, such as charge, polarity, and hydrophobic groups. This feature captures proximal interactions between amino acid groups that can significantly influence the peptide structure and function. Additionally, the CKSAAGP descriptors were normalized by the peptide length to ensure consistency across sequences of varying lengths.

The physicochemical properties: GRAVY index, Molecular Mass, Isoelectric Point, Hydrophobicity and Net Charge were calculated using Biopython library (Cock *et al.* 2009). Finally, the Atomic Composition was calculated by determining the carbon, hydrogen, nitrogen, oxygen, and sulfur quantities and normalized by chain length.

Due to the large number of descriptors, an exploratory data analysis was conducted using the Mann-Whitney statistical test to determine whether each feature showed a significant difference (*P* value <.05) between the CPP and non-CPP groups. As a result, the initial set of 8831 descriptors was reduced to 1137. This is available in Supplementary Subsection 2.1, available as supplementary data at *Bioinformatics Advances* online.

### 2.3 Evaluation strategy

#### 2.3.1 Model training

We applied ERT, Random Forest (RF), XGBoost (XGB), Support Vector Machine (SVM), and Multilayer Perceptron (MLP) algorithms to our training dataset (PerseuCPP training dataset) to identify the one that would produce the best results. Additionally, a Singular Value Decomposition (SVD) was applied to perform dimensionality reduction and the model performance was evaluated considering up to 100 singular values. We conducted a 10-fold cross-validation yielding an average performance that reflects the model stability across different training and testing data configurations.

#### 2.3.2 Comparison with alternative methods

We evaluate PerseuCPP and compare it with state-of-the-art methods using the same set of metrics as the competitors such as Sensitivity (SN), Specificity (SP), Accuracy (ACC), Matthews correlation coefficient (MCC), F1, and Area Under the Receiver Operating Characteristic Curve (AUC). The presented metrics are explained and mathematically defined in the Subsection Comparison with alternative methods in Supplementary Subsection 2.2, available as supplementary data at *Bioinformatics Advances* online.

## 3 Results and discussion

To demonstrate our method’s ability to predict CPPs, we conducted a comprehensive set of experiments. First, we evaluated five different algorithms (ERT, RF, XGB, SVM, and MLP) to determine which would be the best for building the model. After selecting the best algorithm, we compared our model with five other predictors, including the state-of-the-art. Additionally, we devised an efficiency predictor for CPPs and compared it with the state-of-the-art method.

### 3.1 PerseuCPP cross-validation results

First, we assessed which algorithm (ERT, RF, XGB, SVM, and MLP) would perform best in the classification of CPPs. We applied the same training matrix for each algorithm and all of them were evaluated using a 10-fold cross-validation. We tested different configurations of the dataset, keeping the same data and descriptors but applying different treatments to each. The results of these analyses are provided in the Supplementary Section 3.1, available as supplementary data at *Bioinformatics Advances* online.

The ERTs algorithm exhibited the best performance and was crucial for selecting the most informative descriptors, thus highlighting the importance of each descriptor. From the original set of 1,137 descriptors, we reduced the dimensionality to 522, as detailed in Table S12 of Supplementary Subsection 3.2, available as supplementary data at *Bioinformatics Advances* online.

### 3.2 PerseuCPP results compared with state-of-the-art methods

#### 3.2.1 CPP924 validation dataset

In this section, the performance of PerseuCPP was compared with five CPP predictors: CPPred-RF, TargetCPP, StackCPPerd, PractiCPP, and SiameseCPP. The comparisons were conducted using the CPP924 validation dataset to evaluate the performance of our method regarding existing predictors. This dataset is balanced between CPPs and non-CPPs, comprising 924 natural peptides, all of which have been experimentally validated.

SiameseCPP is a deep-learning architecture that learns discriminative descriptors directly from peptide sequences (Zhang *et al.* 2023). These descriptors were fused with contrastive descriptors to achieve good classification performance. The method was built with CPP924 dataset, in which 80% was used to train and 20% to test and was compared with competitors. Despite generally presenting good results, the model lacks interpretability and practical biological insights.

According to (Ramezan *et al.* 2019), building and testing a classification model with 80% of the data used to train and 20% to test is less robust than cross-validation, and there is a possibility that the results obtained in this way could be overestimated. However, for fair comparison, we used the same partition with 20% of the dataset to test our method against competitors, as it was the same configuration used by the then state-of-the-art method. Table 1 presents the comparison between PerseuCPP and the competing methods on CPP924. Our strategy presents results that are superior to SiameseCPP on the CPP924 dataset.

**Table 1.** Comparison of PerseuCPP results with competing methods on dataset CPP924.^a^

Method	MCC	ACC	SN	SP	AUC
PerseuCPP20	0.745	0.878	0.764	0.844	0.933
PerseuESM	0.869	0.934	0.894	0.926	0.973
CPPred-RF	0.831	0.916	0.905	0.926	–
TargetCPP	0.871	0.935	0.943	0.936	–
StackCPPred	0.890	0.945	0.942	0.948	–
PractiCPP	0.913	0.956	0.943	0.971	–
SiameseCPP	0.923	0.961	0.959	0.964	–
**PerseuCPP**	**0.940**	**0.970**	**0.961**	**0.978**	0.975

a The bold values denote the best performance among state-of-the-art methods. AUC values were not reported by the authors of the compared methods.

PractiCPP (Shi *et al.* 2024) employs hard negative sampling to enhance decision boundaries and utilizes three feature types: sequential descriptors from amino acid sequences, local descriptors derived from Morgan fingerprints [which capture the chemical environment around each atom (Morgan 1965)], and pretrained embeddings from the ESM-2 language model. PractiCPP outperforms state-of-the-art models like SiameseCPP and MLCPP2.0 on both balanced and imbalanced datasets. On the other hand, our model outperforms PractiCPP on the CPP924 dataset, Table 1. Nevertheless, it was not possible to undertake a fair comparison using the imbalanced dataset, as it was not provided alongside the source code in the PractiCPP article.

#### 3.2.2 MLCPP2.0 validation dataset

In this section, the performance of PerseuCPP was compared with four CPP predictors: C2Pred, BchemRF, MLCPP2.0, and SiameseCPP. For this purpose, the MLCPP2.0 validation dataset was employed, which was likewise utilised as a benchmark by the aforementioned four methods. This dataset comprises 157 CPPs (experimentally validated) and 2184 non-CPPs (generated artificially).

The results on Table 2 demonstrate that PerseuCPP outperforms the methods C2Pred, BchemRF, and MLCPP 2.0, as well as the SiameseCPP. This superiority highlights the effectiveness of PerseuCPP and its potential practical application in CPP predictions.

**Table 2.** Comparison of PerseuCPP results with competing methods on dataset MLCPP2.0.^a^

Method	MCC	ACC	SN	SP	AUC
C2Pred	0.326	0.781	0.790	0.781	0.867
BchemRF	0.467	0.893	0.745	0.908	0.914
MLCPP2.0	0.624	0.934	0.847	0.940	0.928
SiameseCPP	0.652	0.959	0.624	0.983	0.980
PerseuESM	0.794	0.970	0.904	0.973	0.969
PerseuCPP20	0.736	0.974	0.871	0.979	0.972
**PerseuCPP**	**0.854**	**0.989**	**0.860**	**0.994**	**0.984**

a PerseuCPP20 was trained with top 20 descriptors as shown in Fig. 2. PerseuESM was trained in our main dataset and with ESM descriptors. The bold values denote the best performance among state-of-the-art methods.

#### 3.2.3 CPP1708 validation dataset

In this section, the performance of PerseuCPP was compared with four CPP prediction methods: CellPPD, C2Pred, MLCPP2.0, and GraphCPP. This dataset comprises a diverse set of 90 CPPs and 98 non-CPPs, designed to test the generalization ability of predictive models.

GraphCPP (Imre *et al.* 2025) leverages graph neural networks (GNNs) to predict CPPs by modeling peptide sequences as molecular graphs. This method captures intricate relationships between amino acids and their physicochemical properties. By integrating node and edge descriptors, GraphCPP provides robust embeddings that enhance the accuracy of CPP prediction.

To evaluate the robustness of our model, we also trained PerseuCPP on the CPP1708 training set, which contains 854 experimentally validated CPPs and 854 non-CPPs; the results are shown in Table 3 under the label “PerseuCPP*” Additionally, to capture higher-level semantic information for both our main training dataset (“PerseuESM”) and the CPP1708 training set (“PerseuESM*”), we generated protein language embeddings using the ESM1-T6-43M-UR50S model by averaging the token representations from its sixth layer, thus producing a fixed-length vector for each sequence. While this approach considerably increased the dimensionality of the feature space (from 522 to 1289 descriptors, including 767 ESM-derived descriptors), we applied Singular Value Decomposition (SVD) to iteratively test and determine the optimal number of components. Ultimately, a reduction to 43 components provided the best predictive performance on the test set, as reported in Table 3.

**Table 3.** Comparison of PerseuCPP results with competing methods on dataset CPP1708.^a^

Method	MCC	ACC	PR	SN	F1	AUC
CellPPD	0.0000	0.59	0.0	0.0	0.0	–
C2Pred	0.0540	0.571	0.458	0.256	0.328	0.6500
MLCPP2.0	0.3001	0.667	0.786	0.256	0.386	0.6860
GraphCPP	0.5787	0.795	0.776	**0.731**	**0.752**	0.8459
PerseuCPP*	0.536	0.780	0.775	0.705	0.733	0.864
PerseuESM	0.652	0.811	0.979	0.595	0.740	0.853
PerseuESM*	0.526	0.766	0.743	0.743	0.738	0.891
PerseuCPP20	0.515	0.754	0.846	0.556	0.671	0.849
**PerseuCPP**	**0.646**	**0.811**	**0.960**	0.607	0.744	**0.8652**

a PerseuCPP* represents PerseuCPP trained in CPP1708 training dataset, PerseuESM was trained in our main dataset and with ESM descriptors, and PerseuESM* was trained in CPP1708 training dataset with ESM descriptors. The bold values denote the best performance among state-of-the-art methods.

The results presented in Table 3 demonstrate that PerseuCPP showing compatible or superior results in comparison with the state-of-the-art competitor, as presented in Table 3, showcasing its robust prediction capability even under challenging conditions.

#### 3.2.4 Summary of comparison with state-of-the-art methods

Overall, the version of PerseuCPP trained with the selected 522 traditional descriptors consistently achieved the best performance across all three independent test datasets, MLCPP2.0, CPP924, and CPP1708, when compared with state-of-the-art models and our own ESM-enhanced models. Notably, although the ESM-based version (PerseuESM) performed well, especially on the MLCPP2.0 and CPP924 datasets, it did not surpass the 522-feature model on any of the three benchmarks. This consistency highlights the robustness and generality of the PerseuCPP model built on physicochemical, structural, and atomic descriptors, which are both biologically interpretable and computationally efficient. These results support the decision to adopt this configuration as the main version of the model, offering a favorable balance between performance, interpretability, and practicality.

To further support the comparative evaluation, we performed Wilcoxon signed-rank tests comparing PerseuCPP with the best competing method across the independent datasets. The results did not show statistically significant differences (P>.05), indicating that PerseuCPP performs competitively across multiple benchmarks, a detailed comparison is provided in the Supplementary Material, Subsection 3.3, Table 13, available as supplementary data at *Bioinformatics Advances* online. However, it is important to note that these tests are based only on average values reported for the competing models, since replicate-level results were not available. Therefore, while more robust statistical comparisons were not feasible, our approach remains consistent with the evaluation practices commonly adopted in the field, where previous state-of-the-art models have also been compared in a descriptive manner rather than formal statistical significance testing. Finally, a case study was conducted to illustrate the behavior of the proposed model in real cases of peptides that were recently discovered, which is presented in Supplementary Material, Subsection 3.4, available as supplementary data at *Bioinformatics Advances* online.

### 3.3 Feature importance

The first part of this study was focused on the effects of combining physicochemical, structural, and atomic characteristics on the classification of CPPs. The algorithm Extra Trees was instrumental in this, providing insights into the importance of each feature. Figure 2 presents the top 20 descriptors, underscoring their relevant role in the classification process.

**Figure 2.**
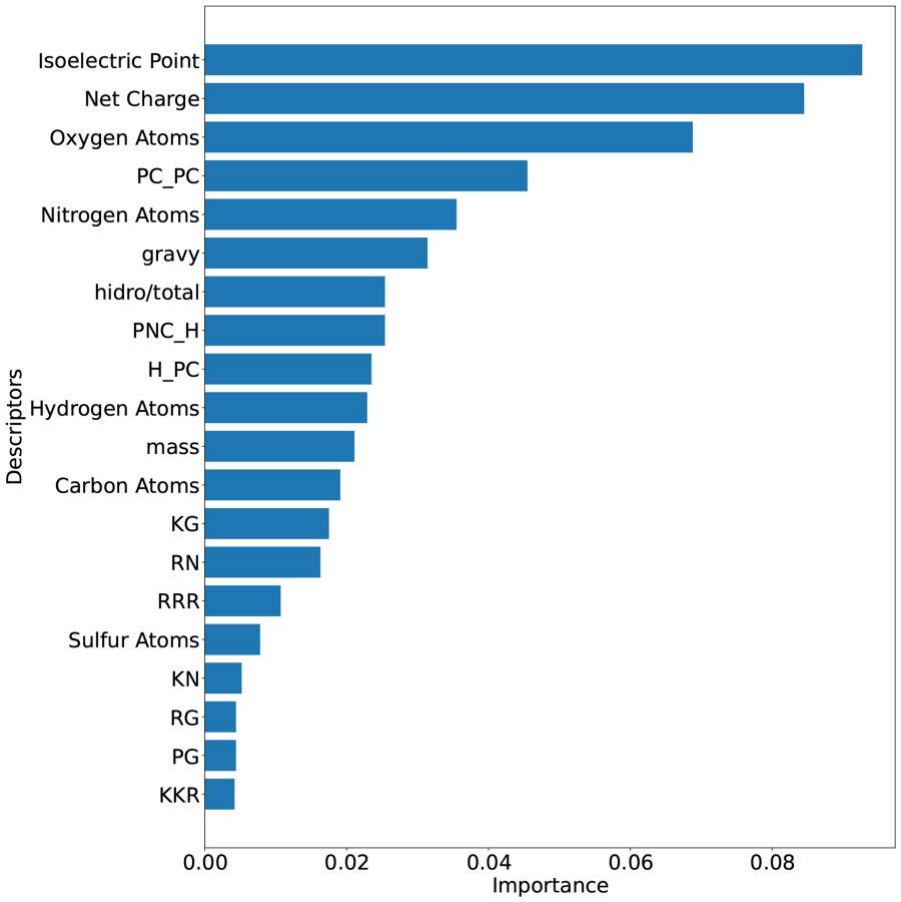
Top 20 independent descriptors for CPP classification identified by Extremely Randomized Trees algorithm. The abbreviations PC, PNC, and H represent the amino acid groups: charged, polar non-charged, and hydrophobic, respectively.

**Figure 3.**
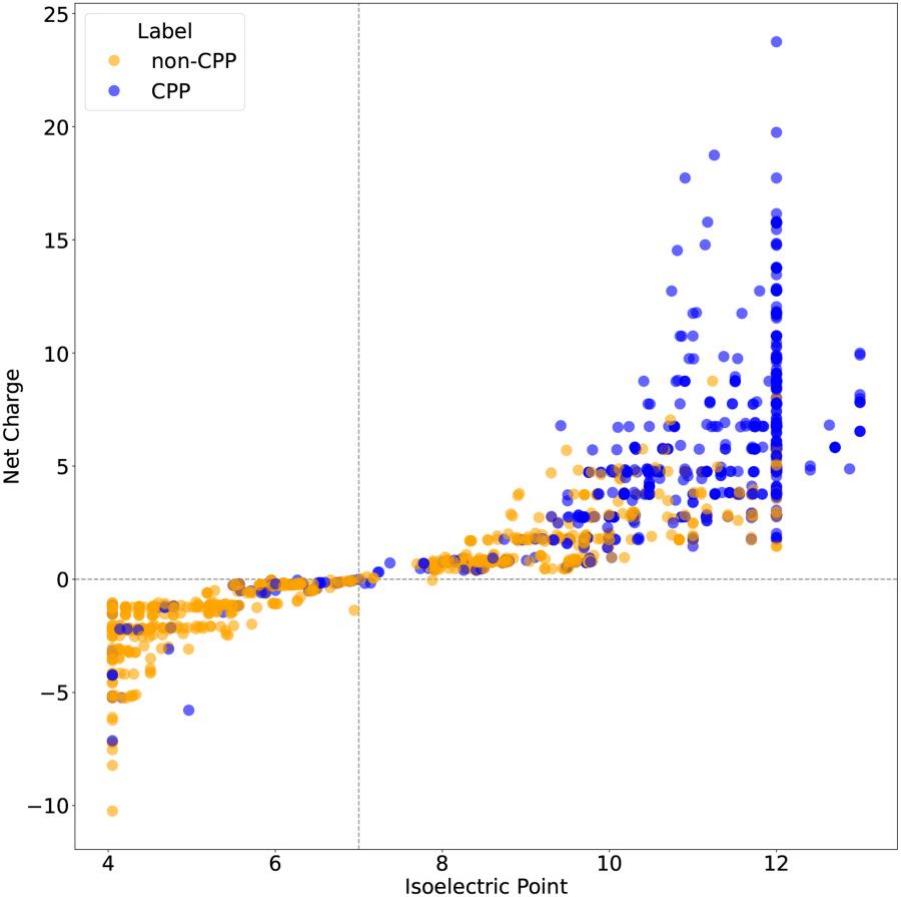
Scatter plot of CPPs and non-CPPs based on isoelectric point and net charge on training dataset. CPPs are predominantly cationic peptides, showing values of isoelectric point superior to 7 under physiological conditions, while a large number of non-CPP are anionic peptides, with values of isoelectric point inferior to 7. The dotted line at x=7 indicates the isoelectric point, highlighting where the charge transition begins.

The Isoelectric Point and Net Charge are descriptors in the group of physicochemical properties and are highly influential in the characterization of CPPs. In Fig. 3, it was confirmed that the analysed CPPs predominantly presented values of isoelectric point greater than seven (pI>7), thus being cationic in their vast majority under physiological conditions. According to the literature, the cell membrane is primarily composed of phospholipids, that could show the phosphate groups and substituent groups negatively charged in their polar heads (Alberts *et al.* 2002), facilitating the interaction of CPPs with cell membranes, especially in bacteria, confirming the relevance of these characteristics in the classification of CPPs.

These top 20 descriptors in Fig. 2 are sufficient for the classification of CPPs, even though there is a low presence of non-CPP peptides with high isoelectric points (cationic) and CPPs with isoelectric points below seven and a negative charge, as shown in Fig. 3. The model trained exclusively with these 20 descriptors demonstrates reasonable performance compared to competitors as shown in Table 2. However, incorporating new descriptors is necessary to ensure better data separation.

Studies suggest that a balance among physicochemical characteristics is critical for a peptide to interact with cellular membranes (Desale *et al.* 2021, Allen and Pellois 2022). This behavior is observed in the model, as besides Isoelectric Point and Net Charge, Gravy, Hydrophobicity, and Mass occupy positions 6, 7, and 11 in importance in Fig. 2.

**Figure 4.**
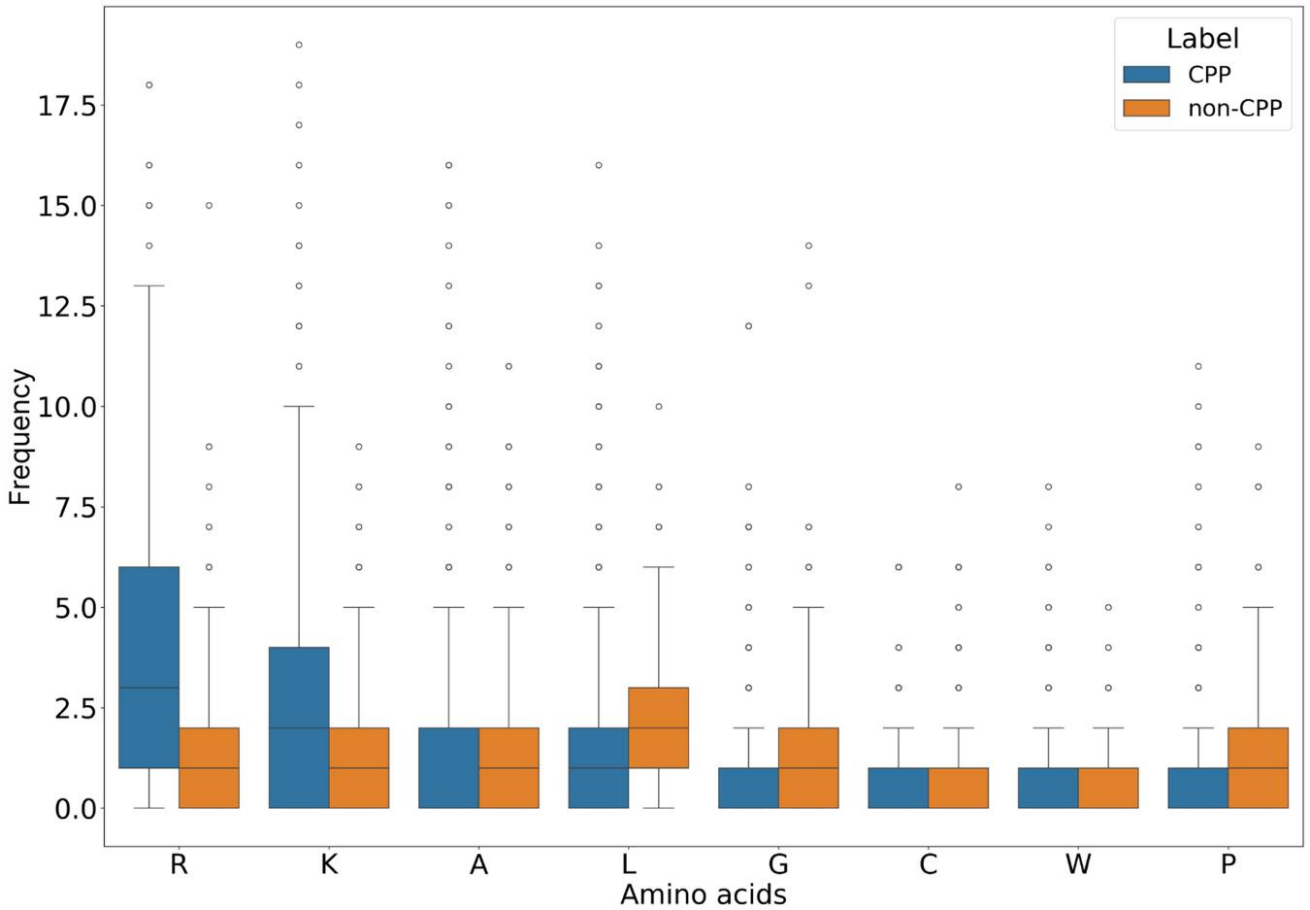
Boxplot comparing the quantity of different amino acids. The x-axis represents the amino acids, while the vertical y-axis indicates the frequency of these amino acids in each sequence. The boxes display the median, quartiles, and outliers for each group.

Arginine (R) and lysine (K) are among the residues relevant for the effectiveness of CPPs, since they are cationic. Arginine-rich CPPs hold promise for delivering therapeutic macromolecules such as peptides, proteins, and nucleic acids into cells (Schmidt *et al.* 2010). The significance of arginine in CPPs is underscored by its involvement in electrostatic interactions and its contribution to the binding affinity of CPPs to cell membranes (Schmidt *et al.* 2010). Furthermore, lysine-rich peptides have demonstrated higher cell-penetrating activity compared to other peptides (Kim *et al.* 2018). It is, therefore, reasonable that the model identified arginine and lysine as critical descriptors for classification, as shown in Fig. 2. These amino acids are highlighted in dipeptides KG, RN, KN, and RG at positions 13, 14, 17, and 18, respectively, as well as in tripeptides RRR and KKR at positions 15 and 20. Based on this, we also analysed the presence of each amino acid in peptide chains. Corroborating this information, Fig. 4 shows the frequency of each amino acid in peptide chains, with arginine and lysine appearing in greater abundance in chains experimentally classified as CPPs.

**Figure 5.**
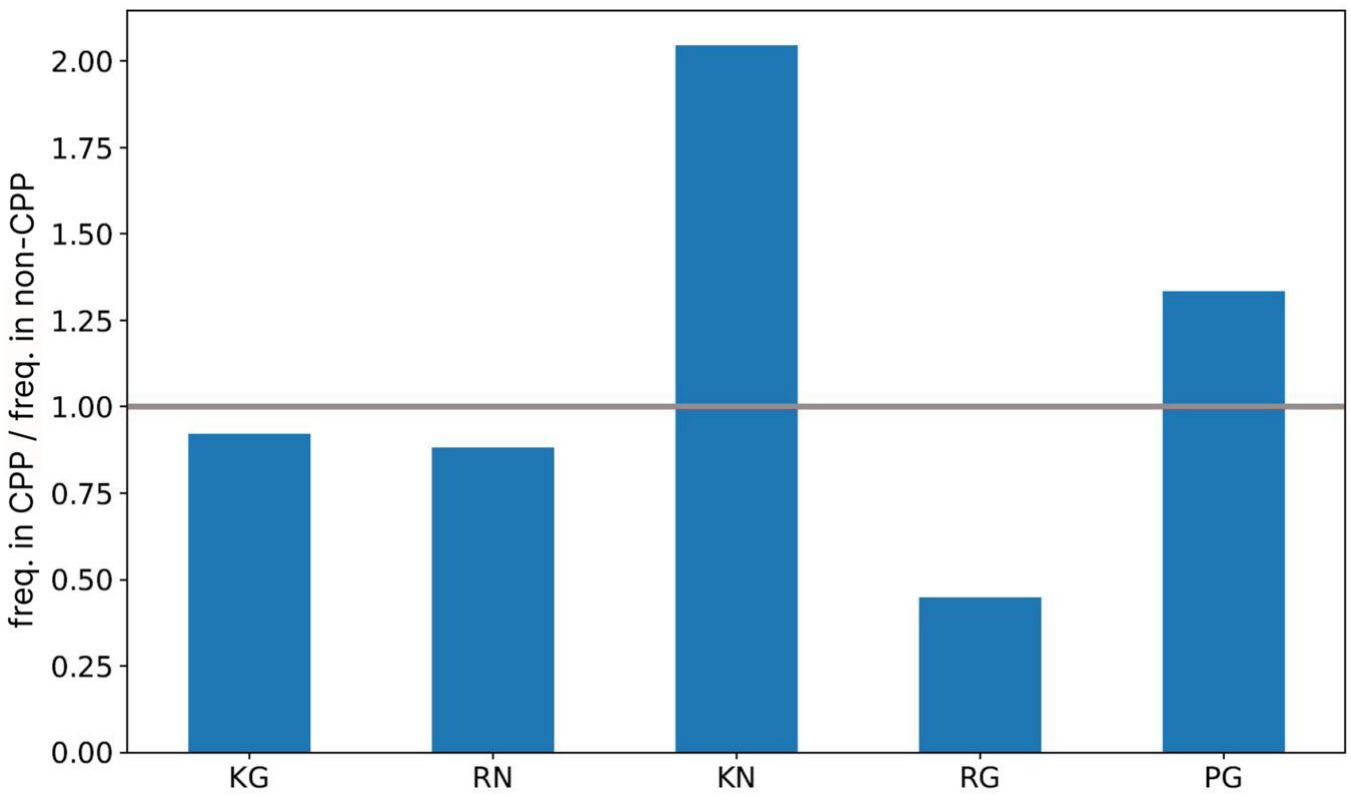
The ratio of the frequencies of the descriptors KG, RN, KN, RG, and PG between the groups CPP and non-CPP. Each bar in the chart represents the ratio (frequency in CPP/frequency in non-CPP) of a specific feature, calculated by dividing the frequency of that feature in the CPP group by its frequency in the non-CPP group. Values greater than 1 indicate that the feature is more frequent in the CPP group, whereas values less than 1 indicate a higher frequency in the non-CPP group.

The utility of dipeptide composition extends to predicting subcellular protein localization (Li and Gao 2020), identifying bacterial toxin proteins (Su *et al.* 2014), and designing highly efficient CPPs (Gautam *et al.* 2013). Particularly in the search for CPPs, dipeptide composition has been employed to differentiate between CPPs and non-CPPs (Gautam *et al.* 2013). As can be observed in Fig. 5, the CPPs/non-CPPs ratio for the dipeptides KG, RN, KN, RG, and PG suggests that the presence of KN and PG is more common in CPPs, while KG, RN, and RG are more present in non-CPPs.

Each amino acid residue comprises a certain amount of carbon (nC), hydrogen (nH), nitrogen (nN), oxygen (nO), and sulfur (nS) in its composition. In this work, the frequency of these atoms normalized by chain length are used as descriptors. It is observed in Fig. 2 that the descriptors Oxygen Atoms and Nitrogen Atoms play an important role in classification. The relationship between the number of nitrogen and oxygen atoms and the cell-penetrating capabilities of peptides has been investigated, highlighting the significance of these atoms in enhancing peptide function (Kalafatovic and Giralt 2017, Hao *et al.* 2022). Specifically, arginine-rich peptides, which contain multiple nitrogen atoms in their guanidinium groups, have been shown to penetrate cell membranes effectively. This is primarily due to their ability to form hydrogen bonds and engage in electrostatic interactions with negatively charged cell membrane components, such as phospholipids and glycosaminoglycans (Hao *et al.* 2022).

Moreover, the presence of oxygen atoms in specific peptides, often in the form of hydroxyl groups or carbonyl groups, can contribute to their amphipathic nature, which is essential for the membrane translocation process. These groups facilitate interactions with the membrane’s hydrophilic and hydrophobic regions, thereby improving the peptide ability to cross the lipid bilayer (Kalafatovic and Giralt 2017).

**Figure 6.**
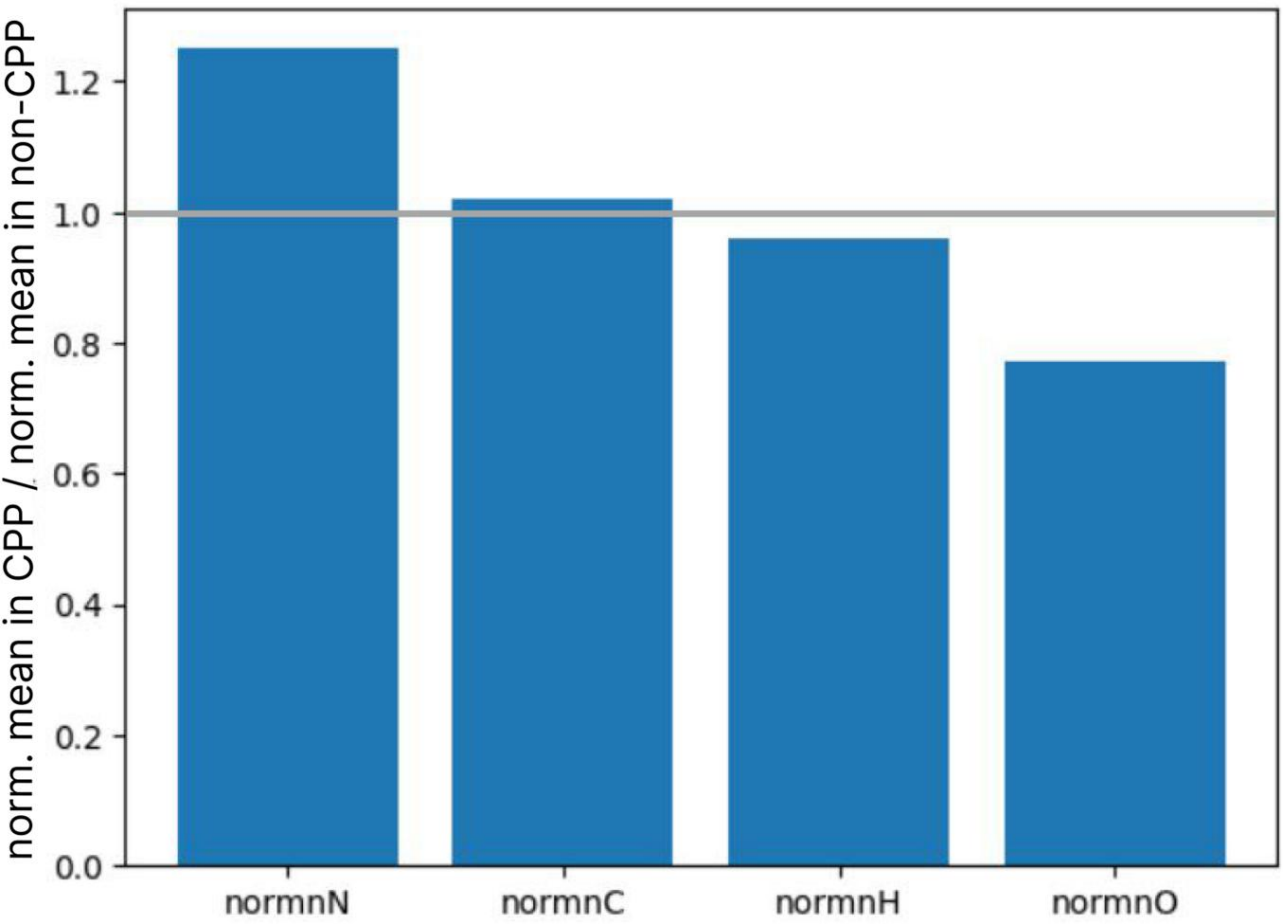
The ratio of the normalized means of the descriptors normnN, normnO, normnH, and normnC between the groups CPP and non-CPP. Each bar in the chart represents the ratio (normalised mean in CPP/normalised mean in non-CPP) of a specific feature, calculated by dividing the normalised mean of that feature in the CPP group by the corresponding value in the non-CPP group. Values greater than 1 indicate that the normalized mean of the feature is higher in the CPP group, whereas values less than 1 indicate a higher normalised mean in the non-CPP group.

It is worth noting that the amount of nitrogen is higher in CPPs compared to non-CPPs, whereas the amount of oxygen is greater in non-CPPs as shown in Fig. 6. This result is justified by the radicals of amino acid residues that may contain ionizable groups, becoming positive by the protonation of the amino groups (in the presence of nitrogen), or becoming negative by the deprotonation of the carbonyl or hydroxyl groups (in the presence of oxygen) at physiological pH. Thus, cationic peptides are rich in nitrogen, while anionic peptides are rich in oxygen.

As shown in Fig. 2, the descriptors PC_PC, PNC_H and H_PC as part of the CKSAAGP (Composition of k-spaced Amino Acid Group Pairs) with amino acid distance of *S *= 1 also had a strong influence on the classification. This feature captures the frequency of pairs of amino acid groups separated by one residue, reflecting proximal interactions that can directly influence the structure and function of the peptides. Specifically, interactions between positive charges with positive charges (PC_PC), uncharged polar groups with hydrophobic ones (NPC_H), and hydrophobic with positive charges (H_PC) are described in the literature as relevant for the cell penetration capability (Mullard 2023). These interactions affect crucial properties such as cell membrane affinity, conformational stability, and solubility, contributing significantly to the efficiency of peptide penetration.

**Figure 7.**
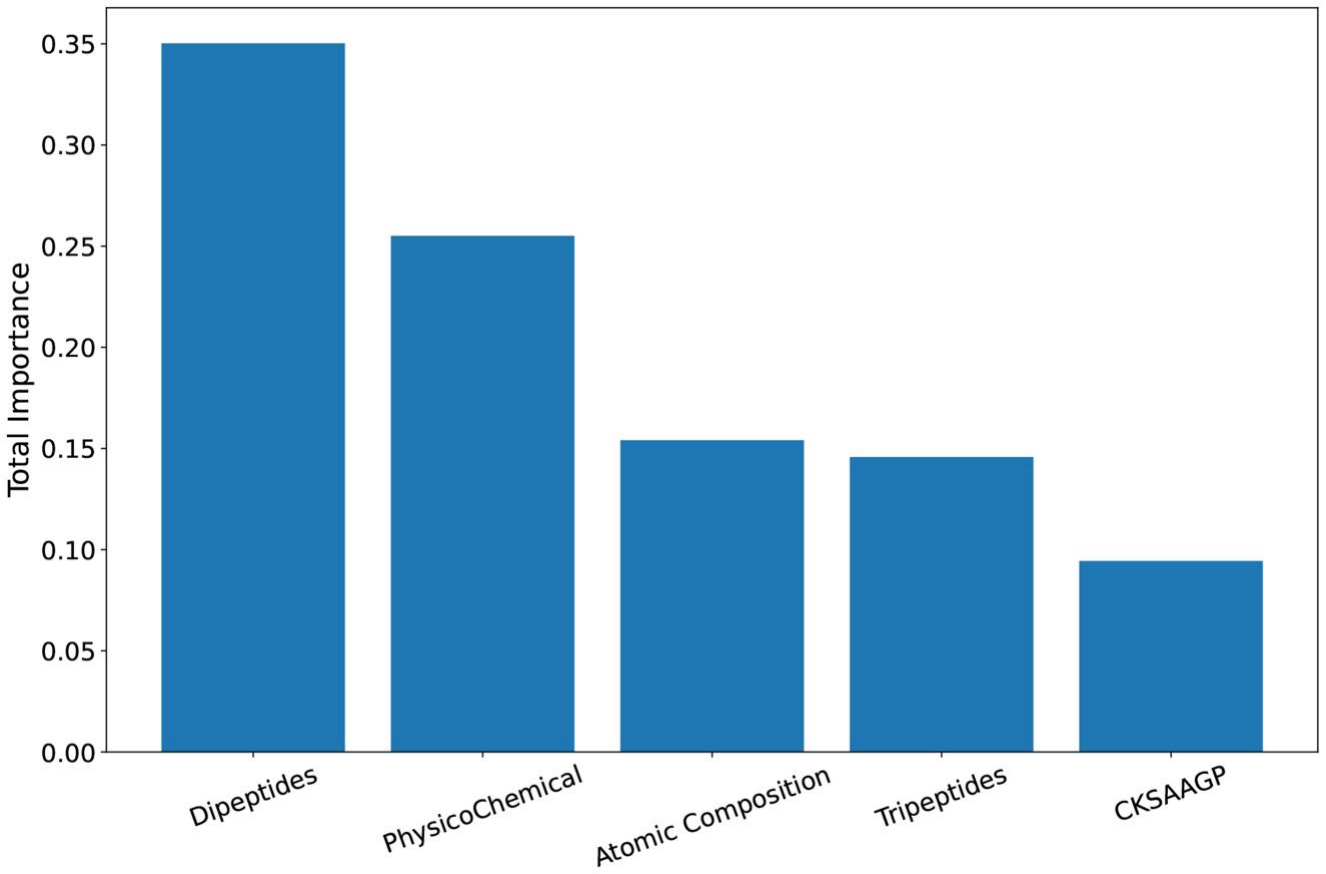
Relative contribution of different groups of descriptors in the classification of CPPs. Each bar represents the contribution of the feature group, in which dipeptides represent groups of two amino acids and tripeptides represent groups of three. The physicochemical group includes descriptors such as GRAVY index (grand average of hydropathicity), molecular mass, isoelectric point, hydrophobicity, and net charge. Atomic composition refers to the atoms of nitrogen, carbon, hydrogen, oxygen, and sulfur. CKSAAGP represents the composition of k-spaced amino acid group pairs.

Figure 7 illustrates the relative contribution of different descriptor groups in the classification of CPPs by PerseuCPP. The analysis reveals that dipeptides and tripeptides have significant influence in the classification task. However, when analysed in isolation, i.e. descriptor by descriptor, they appear to have little influence, as could be expected, considering that there are $20^{2}$ dipeptides and $20^{3}$ tripeptides. Other descriptors have significant impact even when considered in isolation. For instance, the isoelectric point, which is part of the physicochemical group of descriptors, presents an individual contribution of nearly 10%. Also, the other physicochemical descriptors, such as net charge, hydrophobicity, gravy and mass, also play an important role, especially for cationic peptides that interact with anionic regions of phospholipids on the cell surface (Alberts *et al.* 2002). Additionally, atomic composition showed a moderate contribution, suggesting that specific elements, like nitrogen and oxygen, influence electrostatic interactions and hydrogen bonding with membrane components (Kalafatovic and Giralt 2017, Hao *et al.* 2022). Overall, this analysis of descriptors segmented in categories indicates that each group has a relevant contribution for the prediction of CPPs, emphasizing the need for descriptors that perceive physicochemical properties, composition of residues as well as some context (as captured by di and tripeptides), as all of these are connected with CPPs ability to deliver therapeutic molecules.

In Supplementary Subsection 3.2, Fig. 7, available as supplementary data at *Bioinformatics Advances* online, we present the t-distributed Stochastic Neighbor Embedding (t-SNE) visualization, a dimensionality reduction strategy used to visualize complex, high-dimensional data that showed a significant separation between CPPs and non-CPPs.

### 3.4 Incorrect predictions

Considering the independent datasets CPP924, MLCPP2.0 and CPP1708, our model incorrectly predicts 72 in 2699 sequences, with 11 from CPP924, 27 from MLCPP2.0 and 34 from CPP1708. Table 4 shows the number of incorrect predictions for each dataset considering CPPs and non-CPPs.

**Table 4.** Incorrect predictions in PerseuCPP.^a^

Base	Label	Freq.	CP	IP
CPP924	CPP	92	82	10
	non-CPP	93	92	1
MLCPP	CPP	157	145	12
	non-CPP	2170	2155	15
CPP1708	CPP	90	58	32
	non-CPP	96	94	2

a Freq. is the number of sequences in CPP and nonCPP subsets, CP is correct predictions and IP is incorrect predictions.

**Figure 8.**
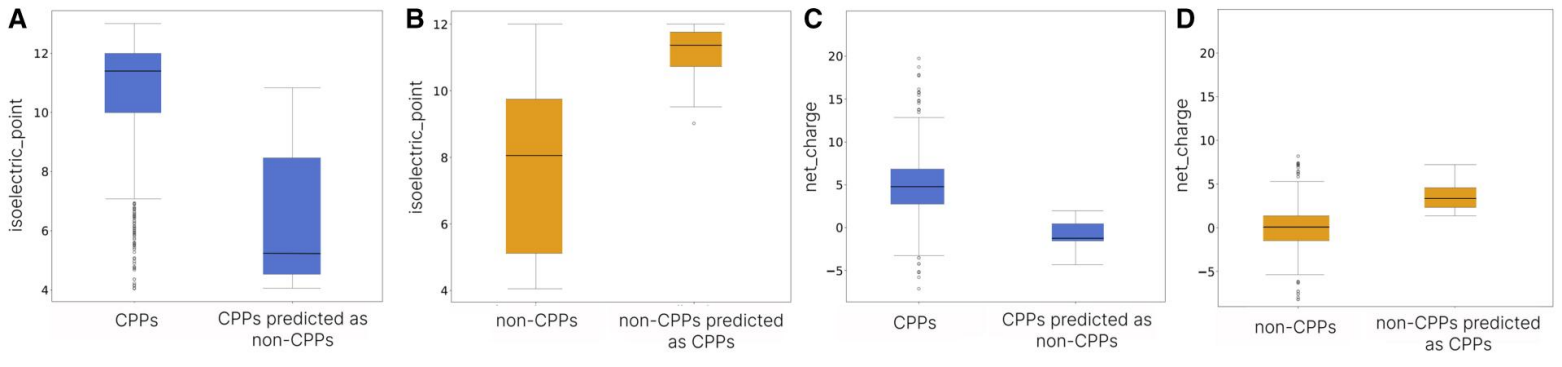
Isoelectric point and net charge comparison of train and misclassified sequences. (A) The distribution of isoelectric points for positive train sequences and misclassified sequences. (B) Isoelectric point distribution for negative train sequences and misclassified sequences. (C) The net charge distribution for positive train sequences and misclassified sequences. (D) The net charge distribution for negative train sequences and misclassified sequences.

To illustrate, here we briefly discuss the descriptors isoelectric_point and net_charge, which are the most important descriptors taken separately. In Fig. 8A, the median isoelectric_point of positive train sequences (blue box labeled as CPPs) has a value close to the median isoelectric_point of the wrongly predicted sequences (orange box labeled as non-CPPs predicted as CPPs) in Fig. 8B. A similar behavior is observed when analyzing the net_charge, as in Fig. 8C the median net charge of positive train sequences (blue box labeled as CPPs) has a value close to the median net charge of wrongly predicted sequences (orange box labeled as non-CPPs predicted as CPPs) in Fig. 8D. Thus, it seems that what causes confusion in our model is that the negative wrongly predicted sequences (non-CPPs predicted as CPPs), have descriptors similar to the positive train sequences.

While ML models may have a tendency to be more sensitive to false positives or false negatives, our model outperformed all the competitors considering the benchmark datasets used in the literature to validate their results. We had instances that were incorrectly predicted in MLCPP2.0 and CPP1708 independent dataset, meaning sequences that should have been classified as negative but were classified as positive. However, this is not necessarily an error due to the nature of the negative sequences. Given the lack of data, many of these sequences were generated automatically based on experimentally validated negative sequences. Thus, these instances could potentially be real positive sequences that have not yet been experimentally determined. In this case, our method would not be making an incorrect prediction, but rather identifying a CPP that has not yet been determined experimentally. In Supplementary Material, Subsection 3.2, Fig. 8, available as supplementary data at *Bioinformatics Advances* online, we show Fig. 3 with the training data, on which we plotted the points that were incorrectly predicted. In addition, we provide on GitHub a table listing the 72 sequences that were incorrectly predicted.

### 3.5 CPP efficiency classifier

The second stage of PerseuCPP was responsible for assessing the efficiency of peptide penetration. In other words, beyond categorizing a peptide as CPP or non-CPP, the proposed method evaluated how efficient the peptide is in terms of cell penetration.

#### 3.5.1 PerseuCPP compared with the state-of-the-art efficiency predictor

To build the PerseuCPP efficiency predictor, we used the atomic composition (the number of carbon, hydrogen, nitrogen, oxygen, and sulfur atoms normalized by chain length). For our method, the atomic composition played an important role in improving results. The MLCPP2.0 efficiency predictor, then considered the state-of-the-art method, focused on descriptors Composition of K-spaced Amino Acid Group Pairs (CKSAAGP), Dipeptide Composition (DPC), and Amino Acid Composition (AAC).

PerseuCPP was successful when predicting CPP efficiency, showing compatible or superior results in comparison with the state-of-the-art competitor, as presented in Table 5.

**Table 5.** Results of PerseuCPP compared with MLCPP2.0 for uptake efficiency prediction.^a^

Method	MCC	ACC	SN	SP	AUC
MLCPP2.0	0.354	0.677	0.652	**0.750**	0.708
**PerseuCPP-Atm**	0.356	**0.726**	**0.761**	0.625	0.690

a Atm represents the atomic composition group of descriptors. Bold values denote the best performance among state-of- the-art methods.

#### 3.5.2 Feature importance

Since we are dealing exclusively with CPPs and attempting to distinguish them by high and low efficiency, a variety of experiments were conducted to evaluate which feature group is capable of improving classification. The results of these experiments are available in the Supplementary Subsection 4.2 in Tables 17 and 18, available as supplementary data at *Bioinformatics Advances* online. The set of descriptors selected to train the model for determining the efficiency of a CPP were the atomic frequencies, as they presented the best results. Figure S9 in Supplementary Subsection 4.1, available as supplementary data at *Bioinformatics Advances* online, provides the detailed importance of each atomic feature.

An important aspect of our model performance is its superior metrics in sensitivity (SN) and accuracy (ACC), and AUC comparable with MLCPP2.0. Our model achieved a SN 0.761, representing a 16.72% increase compared to MLCPP2.0 SN, which is 0.652. SN, which measures the model ability to correctly predict efficient CPPs instances in the context of all the existing positive instances, is a relevant metric for our model as it ensures more accurate identification of efficient CPPs. Additionally, our model presents AUC 0.690, which is close to the value reached by MLCPP2.0.

Although our results for CPP uptake efficiency prediction are promising, it is important to emphasize that the training and testing datasets for this task are still relatively small. As such, we recommend caution when interpreting these results or drawing definitive conclusions until further validation can be performed using larger and more diverse independent datasets.

## 4 Conclusion

Our study introduces PerseuCPP, a ML strategy specifically designed for the prediction of CPPs. By leveraging a comprehensive set of descriptors, encompassing physicochemical properties, atomic characteristics, and sequential descriptors, our model, employing the ERT algorithm, demonstrates superior predictive capabilities.

The thoroughness of our method, reflected in the comprehensive nature of the descriptors, not only provides significant biological insights but also outperforms the state-of-the-art methods across all classification metrics on the MLCPP2.0, CPP924, CPP1708 test datasets, which are the independent datasets considered in the literature for evaluating CPP prediction.

Additionally, there is a second stage of PerseuCPP, which was responsible for assessing the efficiency of peptide penetration, classifying CPPs as low or high efficiency. In this stage, our method presented results comparable or superior to the state-of-the-art.

PerseuCPP introduces a framework that enhances biological interpretability and robustness. The inclusion of experimentally curated sequences, and the feature-driven interpretability of our model enable practical application in research. Moreover, the insights derived from feature analysis may inform future investigations into toxicity, structure-function relationships, and rational peptide design, thus positioning PerseuCPP as a predictive tool and as a method to support deeper biological understanding.

## Supplementary Material

vbaf213_Supplementary_Data

## Data Availability

All the data and source code are available at the GitHub repository: https://github.com/goalmeida05/PERSEU
