# Supplementary material for: PerseuCPP: a machine learning strategy to predict cell-penetrating peptides and their uptake efficiency
Source: Bioinform Adv. 2025 Sep 8;5(1):vbaf213. doi: 10.1093/bioadv/vbaf213 (PMC12462384; doi:10.1093/bioadv/vbaf213)
Supplement: vbaf213_Supplementary_Data [file vbaf213_supplementary_data.pdf]

PERSEUcpp: A machine learning strategy to predict cell-penetrating peptides and their uptake efficiency

Rayane Monique Bernardes-Loch, Gustavo de Oliveira Almeida, Igor Teixeira Brasiliano, Wagner Meira Jr, Douglas E V Pires, Maria Cristina Baracat-Pereira and Sabrina de Azevedo Silveira

August 2025

List of Tables

S1 Performance comparison of machine learning algorithms for CPP prediction using the same dataset. The performance of five machine learning models SVM, XGB, MLP, RF, and ERT in predicting cell-penetrating peptides (CPPs). 13
S2 Performance classification of Extremelly Randomized Trees in independent datasets 13
S3 Performance classification of Random Forest Classifier in independent datasets 14
S4 Performance classification of Multi-Layer Perceptron in independent datasets 14
S5 Performance classification of SVM in independent datasets 14
S6 Performance classification of LGBM in independent datasets . . 15
S7 Performance classification of Gradient Boost in independent datasets 15
S8 Performance classification with SVD and Extremely Randomized Trees in the independent datasets 15
S9 Performance classification with SVD and Random Forest Classifier in the independent datasets 16
S10 Performance classification with SVD and Logistic Regression in the independent datasets 16
S11 Performance classification with SVD and SVM in the independent datasets 16
S12 All descriptors 17
S13 Comparison of PerseuCPP and SiameseCPP across independent datasets with Wilcoxon test p-values. 31
S14 Patented CPP sequences (2023–2025) used for out-of-sample evaluation of PerseuCPP 33

S15 Plant-derived peptides used for out-of-sample evaluation of PerseuCPP. 33
S16 Physicochemical and atomic descriptors for patented and plant-derived peptides. 34
S17 Comparison results of the proposed PERSEUcpp. ALL is all normalized features, TRIPEP is the tripeptides group, DIPEP is dipeptides group, CKSAAGP represents the Composition of k-spaced Amino Acid Group Pairs, PC is the physicochemical group and ATM is the atomic composition group of features . . . 35
S18 Comparison results of the proposed PERSEUcpp with TOP-N features of each descriptors group 35

List of Figures

S1 **Boxplot of CPPs and Non-CPPs based on physicochemical features on training dataset**. CPPs exhibit distinct distributions in features such as hydrophobicity and charge, highlighting their physicochemical differences. 6
S2 **Boxplot of CPPs and Non-CPPs based on CKSAAGP positive charged normalized features on training dataset**. CPPs exhibit distinct distributions in features such as positively_charged_hydrophobic, positively_charged_negatively_charged, positively_charged_polar_non_charged and positively_charged_positively_charged, highlighting their CKSAAGP differences. 7
S3 **Boxplot of CPPs and Non-CPPs based on CKSAAGP polar non charged normalized features on training dataset**. CPPs exhibit distinct distributions in features such as polar_non_charged_hydrophobic, polar_non_charged_negatively_charged, polar_non_charged_polar_non_charged and polar_non_charged_positively_charged, highlighting their CKSAAGP differences. 8
S4 **Boxplot of CPPs and Non-CPPs based on CKSAAGP negatively charged normalized features on training dataset**. CPPs exhibit distinct distributions in features such as negatively_charged_hydrophobic, negatively_charged_negatively_charged, negatively_charged_polar_non_charged and polar_non_charged_positively_charged, highlighting their CKSAAGP differences. 9
S5 **Boxplot of CPPs and Non-CPPs based on physicochemical features on training dataset**. CPPs exhibit distinct distributions in features such as hydrophobicity and charge, highlighting their physicochemical differences. 10
S6 **Boxplot of CPPs and Non-CPPs based on physicochemical features on training dataset**. CPPs exhibit distinct distributions in features such as hydrophobicity and charge, highlighting their physicochemical differences. 11

S7 **t-SNE visualization of CPPs and non-CPPs**. Each point represents a peptide described by a set of calculated descriptors. Points are colored based on their classification: blue for CPPs and orange for non-CPPs. The distinct separation between the blue and orange clusters suggests that the selected features effectively differentiate between CPPs and non-CPPs. 29
S8 **Scatter plot of CPPs and non-CPPs based on isoelectric point and net charge on training dataset**. This figure is the Figure 8 presented in the Feature Importance section of the main paper; however, in addition to the points used in the training to distinguish CPPs from non-CPPs, we have also included the locations of the points that the model misclassified. 30
S9 Top features in importance order of each atomic feature: oxygen, sulfur, nitrogen, hydrogen and carbon atoms. 34

1 Introduction

The CellPPD [1] platform combines ML methods and experimental data to predict CPPs. It focuses on peptide characteristics, including charge, hydrophobicity, and sequence length. Users can submit peptide sequences to the platform, which provides a predictive score indicating the likelihood of cell-penetrating capability based on these features.

CPPpred [2] employs a supervised learning approach to predict the probability of peptides being CPPs. The model uses amino acid composition, physicochemical properties such as molecular weight, isoelectric point, hydrophobicity, and specific sequence motifs commonly found in CPPs. By calculating a score for each peptide, CPPpred allows threshold adjustments to manage false positives.

BChemRF [3], is a ML model for CPP prediction. Using a Random Forest classifier, BChemRF extracts features from peptide sequences, including physicochemical properties, amino acid composition and arrangement, predicted secondary structures, flexibility, and evolutionary information. The ensemble learning approach of BChemRF enhances prediction accuracy and reliability by capturing complex patterns associated with cell-penetrating capability.

SkipCPP-Pred [4] uses a k-skip-n-gram model to convert variable sequence lengths into fixed-length feature vectors. This approach captures the frequency of n residues separated by up to k other residues, including both contiguous and non-contiguous pairs to retain spatial information. The adaptive feature representation adjusts to the length of each peptide, ensuring flexible and inclusive feature extraction. These features are then used to train a ML model to predict the cell-penetrating potential of peptides.

MLCPP2.0 [5], a two-layer classifier named MLCPP2.0 was proposed to predict CPPs and their uptake efficiency. The model uses SVM to predict CPPs based on various features. This predictor achieved superior performance compared to existing predictors and identified independent features for CPP classification. The features used include dipeptide and amino acid compositions, frequency and transition patterns, binary representations, substitution matrices, and physicochemical properties. These groups of features are employed to enhance the accuracy of CPP prediction. The major limitation of this model is that false negatives predicted at the first layer will not be processed by the second layer.

The machine learning model implemented in MLCPP2.0 [5] for predicting the uptake efficiency of cell-penetrating peptides (CPPs) is a meta-model constructed using 11 groups of feature sets. The selected model was the Gradient Boosting with the top 50 feature set. The features utilized included various peptide characteristic encodings such as Amino Acid Composition (AAC), Quasi-Sequence Order (QSO), Composition, Transition, and Distribution (CTDC), Dipeptide Deviation from Expected Mean (DDE), Composition of K-Spaced Amino Acid Pairs (CKS), Dipeptide Composition (DPC), and Composition of K-Spaced Amino Acid Groups Pair (CKSAAGP), among others. These encodings were determined to be important through SHapley Additive exPlanations

(SHAP) analysis, a method that measures the contribution of each feature to the model's predictions based on Shapley values from game theory [6], indicating their role in predicting CPPs in the second layer model.

Recently, the SiameseCPP [7] predictor demonstrated that contrastive learning can extract intrinsic characteristics of CPPs. SiameseCPP is based on deep neural networks, specifically a Siamese network, which learns discriminative features directly from peptide sequences. This method automatically extracts relevant patterns and motifs from the sequences without predefined features, generating high-dimensional representations that capture essential characteristics of the peptides. SiameseCPP improves the accuracy of CPP prediction by identifying complex patterns within the sequences. SiameseCPP achieves superior performance compared to MLCPP2.0, but the study still faces some limitations: of note, lacks interpretability, failing to provide practical biological insights.

PractiCPP [8] is a deep learning framework designed for CPP prediction in highly imbalanced datasets, addressing real-world challenges where positive samples are scarce. The model integrates hard negative sampling to refine decision boundaries and employs three feature types: sequential features from amino acid sequences, local features derived from Morgan fingerprints (a molecular descriptor capturing the local structural environment of atoms [9]), and pre-trained embeddings from the ESM-2 language model. PractiCPP outperforms state-of-the-art models like SiameseCPP and MLCPP2.0 on both balanced and imbalanced datasets, achieving superior metrics such as AUPR and F1 score.

GraphCPP [10] leverages graph neural networks (GNNs) to predict cell-penetrating peptides (CPPs) by modeling peptide sequences as molecular graphs. This method captures intricate relationships between amino acids and their physicochemical properties, outperforming traditional ML models and sequence-based predictors. By integrating node and edge features, GraphCPP provides robust embeddings that enhance the accuracy of CPP prediction. Its superior performance on balanced and imbalanced datasets establishes GraphCPP as a state-of-the-art tool for CPP identification, addressing challenges such as sequence variability and complex molecular interactions.

2 Materials and methods

2.1 Feature engineering

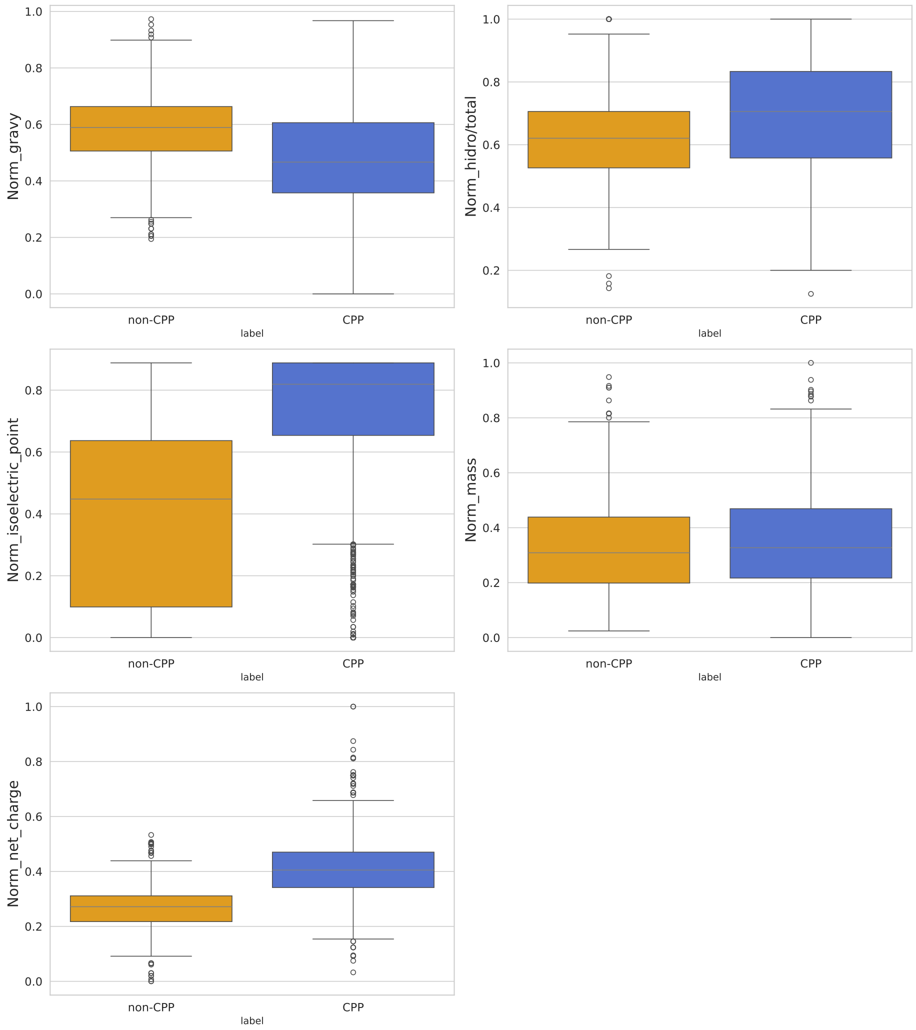

Figure S1: **Boxplot of CPPs and Non-CPPs based on physicochemical features on training dataset**. CPPs exhibit distinct distributions in features such as hydrophobicity and charge, highlighting their physicochemical differences.

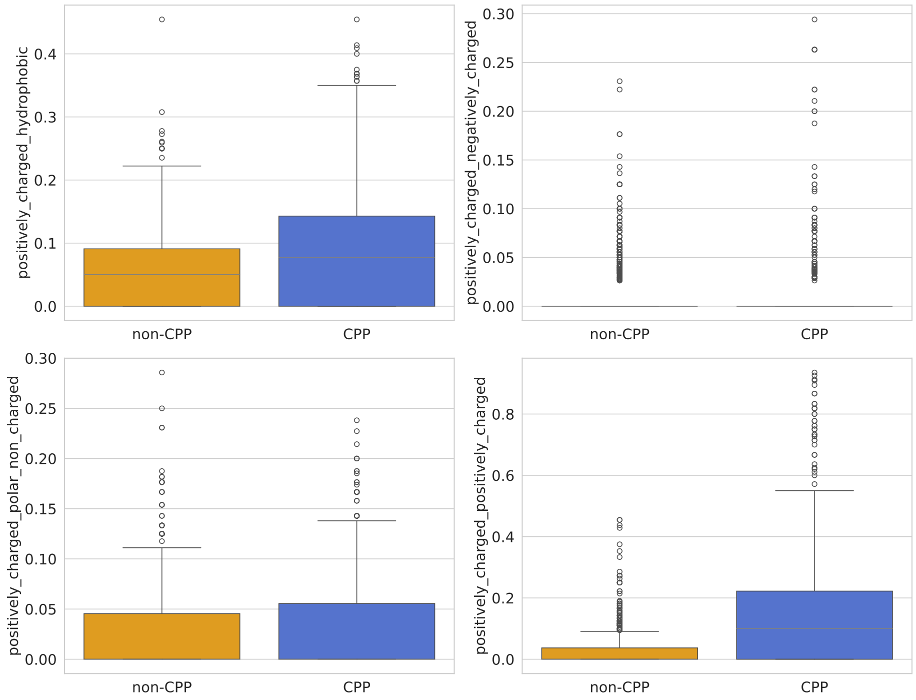

Figure S2: **Boxplot of CPPs and Non-CPPs based on CKSAAGP positive charged normalized features on training dataset**. CPPs exhibit distinct distributions in features such as positively_charged_hydrophobic, positively_charged_negatively_charged, positively_charged_polar_non_charged and positively_charged_positively_charged, highlighting their CKSAAGP differences.

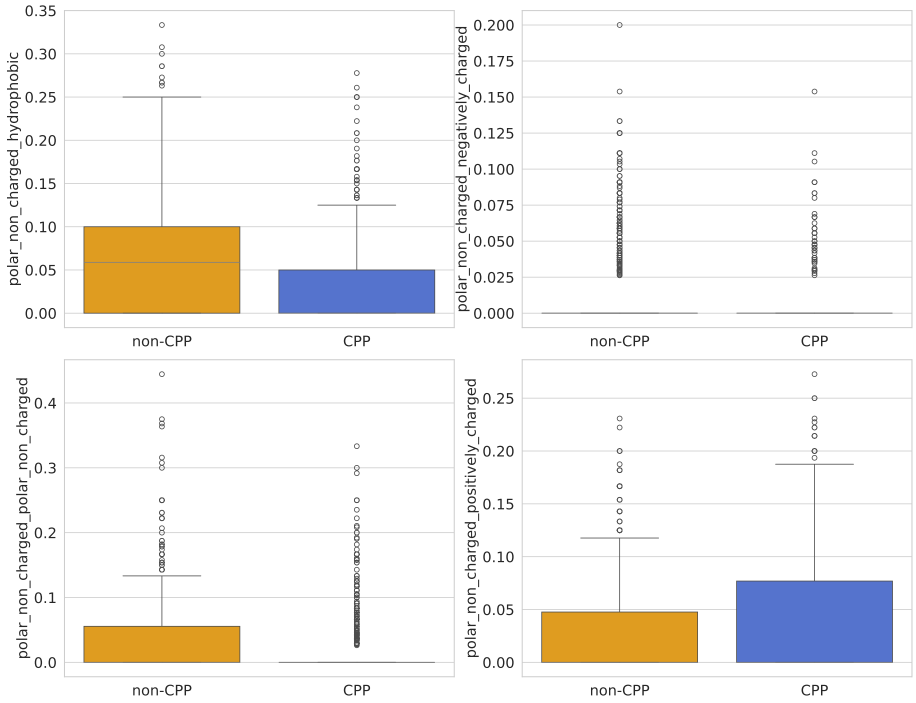

Figure S3: **Boxplot of CPPs and Non-CPPs based on CKSAAGP polar non charged normalized features on training dataset**. CPPs exhibit distinct distributions in features such as polar_non_charged_hydrophobic, polar_non_charged_negatively_charged, polar_non_charged_polar_non_charged and polar_non_charged_positively_charged, highlighting their CKSAAGP differences.

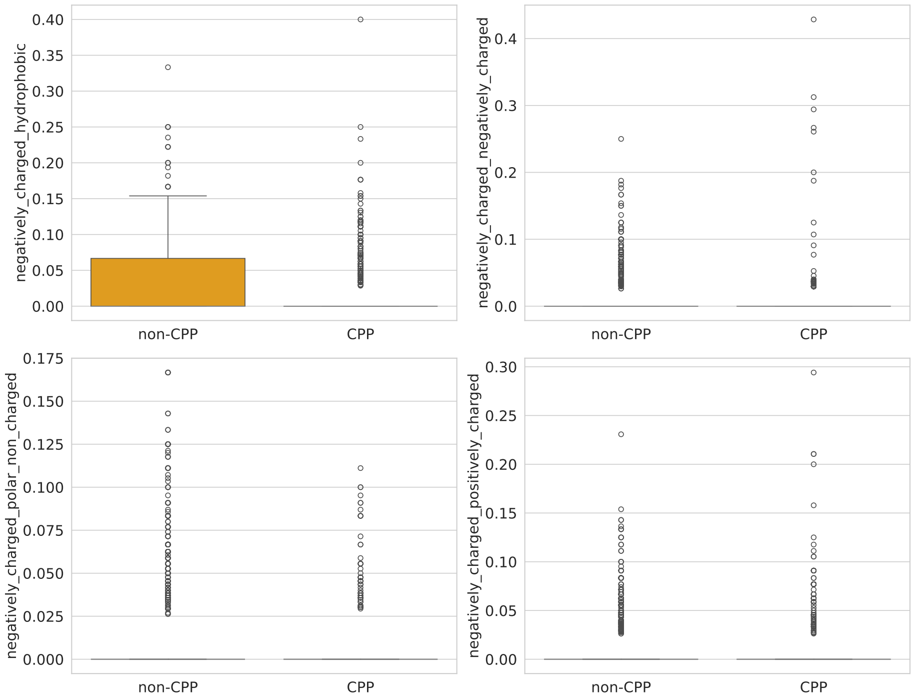

Figure S4: **Boxplot of CPPs and Non-CPPs based on CKSAAGP negatively charged normalized features on training dataset**. CPPs exhibit distinct distributions in features such as negatively_charged_hydrophobic, negatively_charged_negatively_charged, negatively_charged_polar_non_charged and polar_non_charged_positively_charged, highlighting their CKSAAGP differences.

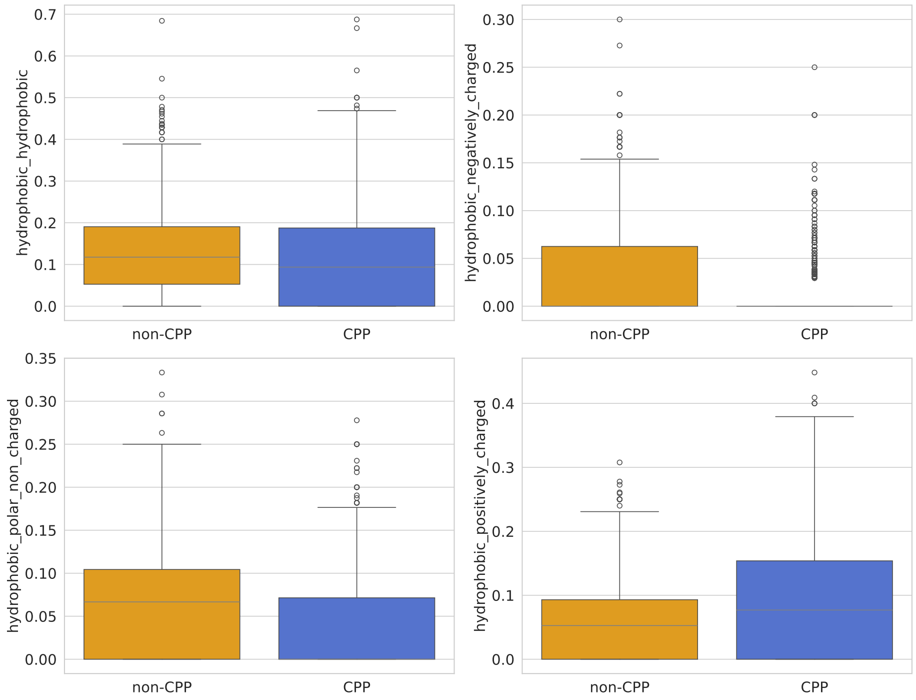

Figure S5: **Boxplot of CPPs and Non-CPPs based on physicochemical features on training dataset**. CPPs exhibit distinct distributions in features such as hydrophobicity and charge, highlighting their physicochemical differences.

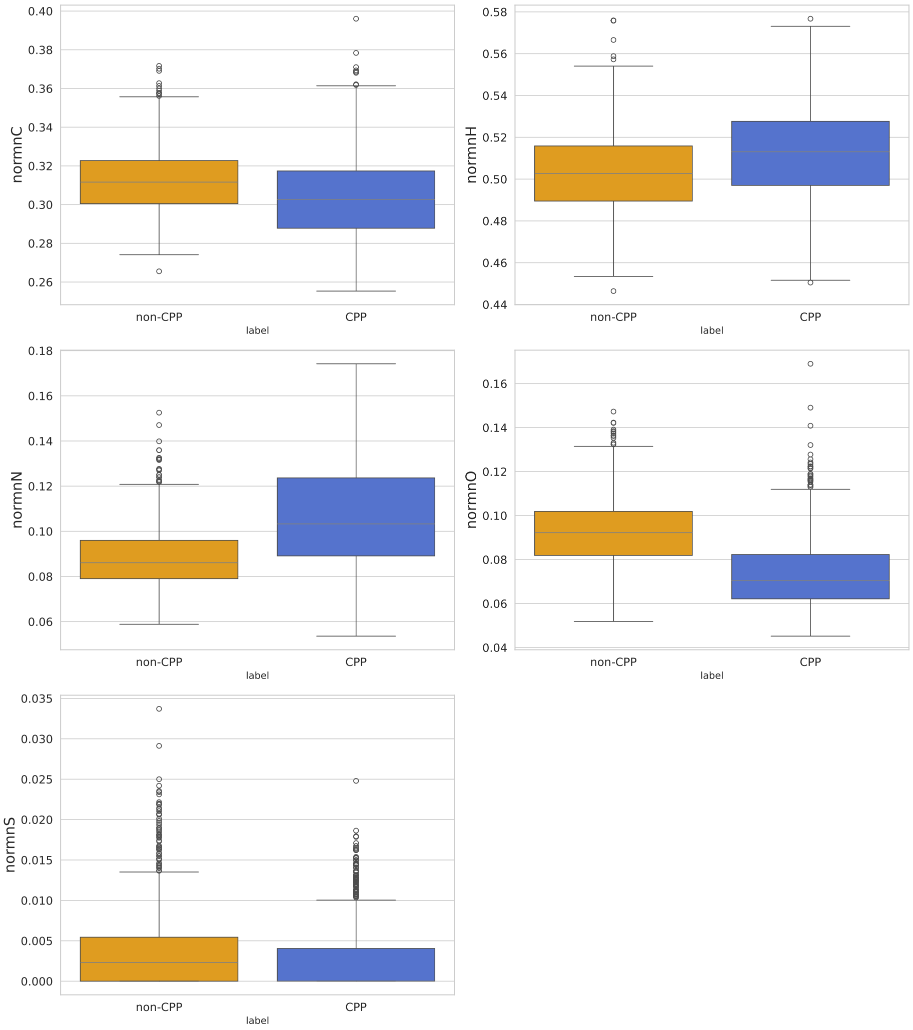

Figure S6: **Boxplot of CPPs and Non-CPPs based on physicochemical features on training dataset**. CPPs exhibit distinct distributions in features such as hydrophobicity and charge, highlighting their physicochemical differences.

2.2 Comparison with alternative methods

Sensitivity, Equation 1, measures the fraction of positive instances correctly identified by the classifier, ranging from 0 to 1. Higher SN means fewer false negatives.

Specificity, Equation 2, measures the fraction of negative instances correctly identified, also ranging from 0 to 1, with higher values indicating fewer false positives.

Accuracy, Equation 3, which measures the overall correctness of the model predictions, also ranges from 0 to 1 but may not be adequate for imbalanced datasets.

The MCC, Equation 4, is a robust measure for binary classification, ranging from -1 (inverse predictions) to +1 (perfect predictions).

The AUC plots the true positive rate against the false positive rate, and represents the model ability to distinguish between classes. An AUC of 0.5 suggests no discriminative ability, with increasing values above 0.5 indicating progressively better discriminative performance. With exception of ACC, all the metrics are robust and appropriate even for scenarios with imbalanced datasets.

TP (True Positive) represents the number of cases correctly classified as positive, while FN (False Negative) refers to the cases that are incorrectly classified as negative despite being positive. FP (False Positive) indicates the number of cases incorrectly classified as positive when they are actually negative, and TN (True Negative) represents the cases correctly classified as negative

$$SN = \frac{TP}{TP + FN} \tag{1}$$

$$SP = \frac{TN}{FP + TN} \tag{2}$$

$$ACC = \frac{TP + TN}{TP + TN + FP + FN} \tag{3}$$

$$MCC = \frac{(TP \times TN) - (FP \times FN)}{\sqrt{(TP + FP)(TP + FN)(FP + TN)(TN + FN)}} \tag{4}$$

3 Results and Discussion

3.1 PERSEUcpp cross-validation results

We tested different configurations of the dataset, keeping the same data and features but applying different treatments to each. The datasets were as follows:

- A matrix in which all the 1,137 features are normalized;
- 99 matrices using the dimensionality reduction technique (SVD) on normalized features, varying the number of singular values from 2 to 100, incrementing by 1.

A large number of features can introduce bias into a machine learning model, mainly if some of these features are not relevant to the classification task. This issue can lead to overfitting, a phenomenon where the model learns patterns that are specific to the training set and fails to generalize effectively to new data. To address this, we used feature importance scores from the best-performing algorithms ERT and RF (Table S1) to identify the most relevant features. Based on this analysis, we reduced the feature set from 1,137 to 522 features. This approach ensured that only the most informative features were retained for further modeling.

Table S1 presents the best results obtained by the algorithms, indicating that the EXT with the top 522 normalized features achieved higher performance in the classification of peptides. Therefore, the ERT algorithm was selected for the next steps. The subsection *PERSEUcpp cross-validation results*, in Tables S2-S11contains the performance data of all the experiments conducted.

Table S1: Performance comparison of machine learning algorithms for CPP prediction using the same dataset. The performance of five machine learning models SVM, XGB, MLP, RF, and ERT in predicting cell-penetrating peptides (CPPs).

Model	MCC	ACC	SN	SP	AUC	F1
SVM	0.192	0.594	0.725	0.466	0.647	0.540
XGB	0.657	0.826	0.769	0.898	0.901	0.872
MLP	0.686	0.841	0.536	0.901	0.906	0.852
RF	0.740	0.896	0.874	0.877	0.949	0.901
**ERT**	**0.801**	**0.934**	**0.868**	**0.998**	**0.981**	**0.926**

Features	MLCPP2.0 Independent Dataset						CPP924 Independent Datasets					
	MCC	ACC	SN	SP	F1-Score	AUC	MCC	ACC	SN	SP	F1-Score	AUC
TOP30	0.662	0.969	0.763	0.978	0.673	0.949	0.765	0.881	0.789	0.956	0.857	0.961
TOP90	0.778	0.981	0.817	0.989	0.787	0.968	0.839	0.917	0.828	0.989	0.900	0.989
TOP150	0.813	0.984	0.860	0.989	0.820	0.979	0.861	0.929	0.855	0.989	0.915	0.992
TOP210	0.829	0.986	0.849	0.992	0.835	0.980	0.883	0.941	0.881	0.989	0.931	0.993
TOP270	0.839	0.987	0.860	0.989	0.836	0.979	0.906	0.952	0.907	0.989	0.945	0.994
TOP330	0.809	0.985	0.860	0.989	0.816	0.980	0.906	0.952	0.908	0.989	0.945	0.991
TOP360	0.837	0.986	0.871	0.990	0.843	0.989	0.929	0.964	0.934	0.989	0.959	0.994

Table S2: Performance classification of Extremelly Randomized Trees in independent datasets

Features	MLCPP2.0 Independent Dataset						CPP924 Independent Datasets					
	MCC	ACC	SN	SP	F1-Score	AUC	MCC	ACC	SN	SP	F1-Score	AUC
TOP30	0.778	0.981	0.86	0.986	0.784	0.979	0.883	0.941	0.882	0.989	0.931	0.995
TOP90	0.805	0.983	0.892	0.987	0.81	0.986	0.917	0.959	0.921	0.989	0.952	0.996
TOP150	0.82	0.985	0.892	0.988	0.826	0.987	0.917	0.959	0.921	0.989	0.952	0.993
TOP210	0.747	0.978	0.817	0.985	0.756	0.977	0.837	0.917	0.842	0.978	0.901	0.984
TOP270	0.828	0.985	0.925	0.987	0.831	0.987	0.890	0.97	0.947	0.989	0.966	0.994
TOP300	0.812	0.984	0.892	0.988	0.818	0.987	0.905	0.953	0.921	0.978	0.946	0.994
TOP330	0.78	0.981	0.871	0.985	0.786	0.978	0.848	0.923	0.855	0.978	0.909	0.986

Table S3: Performance classification of Random Forest Classifier in independent datasets

Features	MLCPP2.0 Independent Dataset						CPP924 Independent Datasets					
	MCC	ACC	SN	SP	F1-Score	AUC	MCC	ACC	SN	SP	F1-Score	AUC
TOP30	0.582	0.957	0.753	0.965	0.588	0.923	0.757	0.876	0.763	0.968	0.847	0.91
TOP90	0.587	0.956	0.774	0.964	0.59	0.933	0.779	0.888	0.789	0.968	0.863	0.938
TOP150	0.566	0.947	0.817	0.953	0.559	0.928	0.777	0.888	0.803	0.957	0.865	0.951
TOP210	0.481	0.937	0.71	0.947	0.482	0.887	0.683	0.84	0.724	0.935	0.803	0.88
TOP270	0.611	0.958	0.806	0.965	0.612	0.922	0.754	0.876	0.776	0.957	0.849	0.944
TOP330	0.415	0.917	0.699	0.926	0.409	0.877	0.669	0.834	0.724	0.925	0.797	0.878

Table S4: Performance classification of Multi-Layer Perceptron in independent datasets

Features	MLCPP2.0 Independent Dataset						CPP924 Independent Datasets					
	MCC	ACC	SN	SP	F1-Score	AUC	MCC	ACC	SN	SP	F1-Score	AUC
TOP30	0.52	0.947	0.71	0.958	0.526	0.902	0.746	0.87	0.75	0.968	0.838	0.921
TOP90	0.532	0.949	0.72	0.959	0.538	0.904	0.746	0.87	0.75	0.968	0.838	0.928
TOP150	0.556	0.952	0.742	0.961	0.561	0.9	0.757	0.876	0.763	0.968	0.847	0.934
TOP210	0.477	0.939	0.688	0.95	0.481	0.891	0.708	0.852	0.737	0.946	0.818	0.902
TOP270	0.554	0.952	0.742	0.961	0.559	0.899	0.768	0.882	0.776	0.968	0.855	0.94
TOP330	0.49	0.941	0.699	0.952	0.494	0.898	0.721	0.858	0.737	0.957	0.824	0.906

Table S5: Performance classification of SVM in independent datasets

Features	MLCPP2.0 Independent Dataset						CPP924 Independent Datasets					
	MCC	ACC	SN	SP	F1-Score	AUC	MCC	ACC	SN	SP	F1-Score	AUC
TOP30	0.746	0.977	0.86	0.982	0.751	0.98	0.917	0.959	0.921	0.989	0.952	0.989
TOP90	0.759	0.977	0.892	0.981	0.761	0.983	0.893	0.947	0.908	0.978	0.939	0.994
TOP150	0.768	0.977	0.925	0.979	0.768	0.985	0.917	0.959	0.921	0.989	0.952	0.996
TOP210	0.601	0.955	0.817	0.961	0.598	0.958	0.822	0.911	0.855	0.957	0.897	0.982
TOP270	0.816	0.984	0.892	0.988	0.822	0.981	0.912	0.976	0.927	0.968	0.974	0.996
TOP330	0.719	0.972	0.882	0.976	0.719	0.97	0.883	0.941	0.882	0.989	0.931	0.99

Table S6: Performance classification of LGBM in independent datasets

Features	MLCPP2.0 Independent Dataset						CPP924 Independent Datasets					
	MCC	ACC	SN	SP	F1-Score	AUC	MCC	ACC	SN	SP	F1-Score	AUC
TOP30	0.668	0.966	0.839	0.971	0.67	0.968	0.859	0.929	0.868	0.978	0.917	0.981
TOP90	0.703	0.969	0.892	0.972	0.7	0.975	0.87	0.935	0.882	0.978	0.924	0.985
TOP150	0.689	0.97	0.839	0.975	0.693	0.971	0.834	0.917	0.868	0.957	0.904	0.983
TOP210	0.638	0.964	0.796	0.971	0.643	0.967	0.822	0.911	0.855	0.957	0.897	0.967
TOP270	0.676	0.967	0.839	0.973	0.678	0.977	0.858	0.929	0.882	0.968	0.918	0.987
TOP330	0.651	0.966	0.796	0.973	0.658	0.968	0.811	0.905	0.842	0.957	0.889	0.971

Table S7: Performance classification of Gradient Boost in independent datasets

Components	MLCPP2.0 Independent Dataset						CPP924 Independent Datasets					
	MCC	ACC	SN	SP	F1-Score	AUC	MCC	ACC	SN	SP	F1-Score	AUC
2	0.167	0.671	0.731	0.669	0.155	0.746	0.801	0.898	0.947	0.857	0.894	0.966
20	0.591	0.945	0.892	0.947	0.572	0.967	0.916	0.958	0.930	0.956	0.954	0.986
40	0.597	0.946	0.889	0.948	0.578	0.969	0.903	0.952	0.934	0.967	0.946	0.987
60	0.602	0.949	0.881	0.952	0.587	0.960	0.915	0.958	0.911	0.978	0.953	0.992
**80**	**0.639**	**0.956**	**0.892**	**0.959**	**0.628**	**0.969**	**0.915**	**0.958**	**0.934**	**0.978**	**0.953**	**0.987**
100	0.631	0.956	0.870	0.960	0.623	0.966	0.867	0.934	0.907	0.956	0.926	0.980

Table S8: Performance classification with SVD and Extremely Randomized Trees in the independent datasets

Components	MLCPP2.0 Independent Dataset						CPP924 Independent Datasets					
	MCC	ACC	SN	SP	F1-Score	AUC	MCC	ACC	SN	SP	F1-Score	AUC
2	0.158	0.679	0.698	0.678	0.152	0.733	0.731	0.862	0.921	0.813	0.858	0.957
20	0.573	0.945	0.849	0.949	0.560	0.952	0.818	0.910	0.881	0.934	0.899	0.962
40	0.572	0.949	0.806	0.955	0.568	0.961	0.761	0.880	0.802	0.945	0.859	0.958
60	0.602	0.949	0.881	0.952	0.587	0.960	0.915	0.958	0.911	0.978	0.953	0.992
80	0.572	0.949	0.806	0.955	0.568	0.959	0.761	0.880	0.802	0.945	0.859	0.960
100	0.609	0.916	0.827	0.956	0.606	0.958	0.832	0.901	0.868	0.956	0.904	0.968

Table S9: Performance classification with SVD and Random Forest Classifier in the independent datasets

Components	MLCPP2.0 Independent Dataset						CPP924 Independent Datasets					
	MCC	ACC	SN	SP	F1-Score	AUC	MCC	ACC	SN	SP	F1-Score	AUC
2	0.064	0.662	0.483	0.669	0.105	0.605	0.616	0.802	0.631	0.945	0.744	0.903
20	0.452	0.935	0.666	0.946	0.457	0.858	0.685	0.844	0.789	0.890	0.821	0.888
40	0.458	0.930	0.709	0.940	0.456	0.885	0.734	0.868	0.815	0.912	0.849	0.892
60	0.465	0.928	0.741	0.935	0.458	0.903	0.760	0.880	0.816	0.934	0.861	0.919
80	0.467	0.928	0.741	0.936	0.460	0.911	0.748	0.874	0.802	0.934	0.853	0.935
100	0.477	0.928	0.802	0.935	0.467	0.914	0.748	0.874	0.802	0.934	0.853	0.947

Table S10: Performance classification with SVD and Logistic Regression in the independent datasets

Components	MLCPP2.0 Independent Dataset						CPP924 Independent Datasets					
	MCC	ACC	SN	SP	F1-Score	AUC	MCC	ACC	SN	SP	F1-Score	AUC
2	- 0.067	0.395	0.440	0.393	0.056	0.417	- 0.044	0.467	0.605	0.351	0.508	0.567
20	- 0.076	0.402	0.408	0.402	0.053	0.434	- 0.044	0.467	0.450	0.360	0.508	0.555
40	- 0.068	0.401	0.430	0.400	0.055	0.434	- 0.044	0.467	0.351	0.265	0.505	0.525
60	- 0.080	0.404	0.397	0.404	0.052	0.433	- 0.059	0.461	0.578	0.362	0.494	0.555
80	- 0.079	0.404	0.397	0.404	0.060	0.555	- 0.059	0.462	0.588	0.352	0.494	0.563
100	- 0.079	0.397	0.403	0.403	0.051	0.434	- 0.055	0.452	0.578	0.383	0.452	0.563

Table S11: Performance classification with SVD and SVM in the independent datasets

3.2 Feature Importance

Table S12: All descriptors

Group	Feature	Importance
PhysicoChemical	Isoelectric Point	0.0927
PhysicoChemical	Net Charge	0.0845
Atomic Composition	Oxygen Atoms	0.0688
CKSAAGP	PC_PC	0.0455
Atomic Composition	Nitrogen Atoms	0.0355
PhysicoChemical	gravy	0.0314
PhysicoChemical	hidro/total	0.0254
CKSAAGP	PNC_H	0.0254
CKSAAGP	H_PC	0.0235
Atomic Composition	Hydrogen Atoms	0.0229
PhysicoChemical	mass	0.0211
Atomic Composition	Carbon Atoms	0.0191
Dipeptides	KG	0.0175
Dipeptides	RN	0.0163
Tripeptides	RRR	0.0107
Atomic Composition	Sulfur Atoms	0.0078
Dipeptides	KN	0.0052
Dipeptides	RG	0.0044
Dipeptides	PG	0.0044
Tripeptides	KKR	0.0042
Dipeptides	LL	0.0041
Dipeptides	AH	0.0041
Dipeptides	PL	0.0041
Dipeptides	PS	0.0038
Dipeptides	LG	0.0038
Tripeptides	VTT	0.0036
Dipeptides	SL	0.0034
Dipeptides	WG	0.0034
Tripeptides	RKK	0.0033
Dipeptides	KV	0.0033
Dipeptides	TQ	0.0032
Tripeptides	KKK	0.0032
Dipeptides	RM	0.0032
Dipeptides	LP	0.0031
Tripeptides	KRR	0.0031
Dipeptides	LH	0.0029
Dipeptides	IT	0.0029
Dipeptides	KH	0.0029
		Next Page

Group	Feature	Importance
Dipeptides	RD	0.0028
Dipeptides	VG	0.0028
Tripeptides	SGS	0.0028
Dipeptides	GN	0.0027
Dipeptides	WN	0.0027
Dipeptides	EA	0.0026
Dipeptides	YN	0.0026
Dipeptides	FV	0.0026
Tripeptides	KRK	0.0026
Dipeptides	GG	0.0026
Dipeptides	LS	0.0026
Dipeptides	PC	0.0025
Tripeptides	CGS	0.0025
Dipeptides	AN	0.0025
Dipeptides	CN	0.0024
Dipeptides	CD	0.0024
Dipeptides	KP	0.0024
Dipeptides	LN	0.0023
Dipeptides	LD	0.0023
Dipeptides	SQ	0.0023
Dipeptides	RF	0.0023
Dipeptides	QY	0.0022
Dipeptides	LM	0.0022
Dipeptides	SD	0.0022
Dipeptides	GH	0.0022
Tripeptides	FPQ	0.0022
Dipeptides	VN	0.0022
Dipeptides	VD	0.0021
Dipeptides	IF	0.0021
Dipeptides	RH	0.0021
Dipeptides	TL	0.0021
Dipeptides	MV	0.0021
Tripeptides	LKK	0.0020
Dipeptides	RE	0.0020
Dipeptides	FN	0.0020
Tripeptides	GSG	0.0019
Tripeptides	TKS	0.0019
Dipeptides	VH	0.0019
Dipeptides	PP	0.0019
Dipeptides	WH	0.0018
Tripeptides	IIY	0.0018
Dipeptides	RQ	0.0018

Next Page

Group	Feature	Importance
Tripeptides	SLQ	0.0017
Tripeptides	IYR	0.0017
Tripeptides	LPP	0.0017
Dipeptides	SP	0.0017
Dipeptides	TP	0.0017
Dipeptides	IV	0.0017
Dipeptides	SN	0.0017
Dipeptides	LA	0.0016
Tripeptides	KAL	0.0016
Dipeptides	RL	0.0016
Dipeptides	NV	0.0016
Dipeptides	FD	0.0016
Dipeptides	AF	0.0016
Dipeptides	KF	0.0016
Tripeptides	MII	0.0016
Dipeptides	YP	0.0016
Tripeptides	SPT	0.0016
Tripeptides	AGY	0.0016
Dipeptides	QW	0.0016
Tripeptides	TFP	0.0016
Dipeptides	SH	0.0016
Tripeptides	LAL	0.0016
Dipeptides	PQ	0.0015
Tripeptides	YSP	0.0015
Dipeptides	TG	0.0015
Tripeptides	DLI	0.0015
Dipeptides	RP	0.0015
Tripeptides	KLA	0.0015
Dipeptides	IP	0.0015
Dipeptides	NP	0.0015
Tripeptides	LLR	0.0015
Tripeptides	GRC	0.0015
Tripeptides	ACI	0.0015
Dipeptides	IG	0.0014
Tripeptides	ALA	0.0014
Tripeptides	PPV	0.0014
Dipeptides	RS	0.0014
Dipeptides	MF	0.0014
Dipeptides	HN	0.0013
Dipeptides	AR	0.0013
Dipeptides	VQ	0.0013
Tripeptides	ADE	0.0013
		Next Page

Group	Feature	Importance
Dipeptides	DR	0.0013
Dipeptides	TN	0.0013
Dipeptides	QH	0.0013
Tripeptides	RDL	0.0013
Dipeptides	KQ	0.0013
Dipeptides	FH	0.0013
Dipeptides	KS	0.0013
Tripeptides	GRR	0.0013
Dipeptides	KD	0.0013
Dipeptides	DV	0.0013
Dipeptides	GM	0.0012
Dipeptides	TS	0.0012
Dipeptides	PH	0.0012
Dipeptides	KA	0.0012
Dipeptides	DF	0.0012
Dipeptides	KY	0.0012
Dipeptides	IH	0.0012
Dipeptides	KR	0.0012
Dipeptides	YH	0.0012
Dipeptides	DE	0.0012
Dipeptides	QV	0.0012
Tripeptides	GYL	0.0012
Dipeptides	RK	0.0012
Tripeptides	RRQ	0.0012
Dipeptides	GP	0.0012
Dipeptides	AP	0.0011
Dipeptides	NN	0.0011
Dipeptides	NM	0.0011
Tripeptides	PPK	0.0011
Dipeptides	QG	0.0011
Tripeptides	LPV	0.0011
Dipeptides	NL	0.0011
Dipeptides	CL	0.0011
Dipeptides	TV	0.0011
Dipeptides	IY	0.0011
Tripeptides	RKV	0.0011
Dipeptides	NH	0.0011
Dipeptides	KT	0.0011
Tripeptides	RQR	0.0011
Dipeptides	WP	0.0011
Dipeptides	IQ	0.0011
Dipeptides	SE	0.0011

Next Page

Group	Feature	Importance
Dipeptides	TT	0.0011
Tripeptides	QRR	0.0011
Dipeptides	PM	0.0011
Dipeptides	LF	0.0011
Dipeptides	IL	0.0011
Tripeptides	TWL	0.0010
Dipeptides	AA	0.0010
Dipeptides	QR	0.0010
Dipeptides	QN	0.0010
Tripeptides	LWE	0.0010
Tripeptides	LAK	0.0010
Dipeptides	EV	0.0010
Tripeptides	AKK	0.0010
Dipeptides	FL	0.0010
Dipeptides	TD	0.0010
Dipeptides	HE	0.0010
Tripeptides	FRI	0.0010
Tripeptides	RCT	0.0010
Dipeptides	IM	0.0010
Dipeptides	WL	0.0010
Tripeptides	RRA	0.0010
Dipeptides	KE	0.0010
Dipeptides	IR	0.0010
Dipeptides	AG	0.0010
Dipeptides	LV	0.0010
Dipeptides	FF	0.0010
Dipeptides	PR	0.0010
Dipeptides	AD	0.0010
Tripeptides	YLL	0.0010
Dipeptides	IC	0.0010
Tripeptides	GCG	0.0010
Dipeptides	WC	0.0010
Dipeptides	AL	0.0010
Tripeptides	RFR	0.0010
Tripeptides	LKA	0.0010
Tripeptides	WLK	0.0009
Dipeptides	NF	0.0009
Dipeptides	LC	0.0009
Tripeptides	YHV	0.0009
Dipeptides	RT	0.0009
Dipeptides	SC	0.0009
Dipeptides	DN	0.0009
		Next Page

Group	Feature	Importance
Tripeptides	RMK	0.0009
Dipeptides	QL	0.0009
Dipeptides	GD	0.0009
Tripeptides	TTE	0.0009
Tripeptides	HLL	0.0009
Dipeptides	KI	0.0009
Dipeptides	IN	0.0009
Dipeptides	SS	0.0009
Tripeptides	QQH	0.0009
Dipeptides	SV	0.0009
Tripeptides	RIL	0.0009
Tripeptides	PSK	0.0008
Dipeptides	PF	0.0008
Dipeptides	AQ	0.0008
Dipeptides	RA	0.0008
Dipeptides	RC	0.0008
Dipeptides	AK	0.0008
Dipeptides	HH	0.0008
Dipeptides	RR	0.0008
Dipeptides	DG	0.0008
Dipeptides	SF	0.0008
Dipeptides	RV	0.0008
Tripeptides	LRP	0.0008
Dipeptides	FY	0.0008
Dipeptides	PD	0.0008
Dipeptides	PN	0.0008
Dipeptides	KM	0.0008
Tripeptides	LCK	0.0008
Tripeptides	RWR	0.0008
Dipeptides	FP	0.0008
Dipeptides	SG	0.0008
Dipeptides	SM	0.0008
Tripeptides	TCN	0.0008
Dipeptides	DH	0.0008
Tripeptides	AAR	0.0008
Dipeptides	EG	0.0008
Dipeptides	GR	0.0008
Dipeptides	GC	0.0008
Dipeptides	NR	0.0008
Tripeptides	SLW	0.0008
Dipeptides	MG	0.0008
Tripeptides	ISH	0.0008
		Next Page

Group	Feature	Importance
Dipeptides	VP	0.0008
Dipeptides	ID	0.0008
Dipeptides	FW	0.0008
Tripeptides	KKI	0.0008
Dipeptides	FK	0.0008
Dipeptides	LR	0.0008
Tripeptides	ARR	0.0007
Dipeptides	KL	0.0007
Tripeptides	YPR	0.0007
Dipeptides	KC	0.0007
Tripeptides	QNR	0.0007
Tripeptides	LKL	0.0007
Dipeptides	ED	0.0007
Dipeptides	IK	0.0007
Dipeptides	VR	0.0007
Dipeptides	GF	0.0007
Tripeptides	KKL	0.0007
Dipeptides	GL	0.0007
Tripeptides	HFR	0.0007
Dipeptides	HY	0.0007
Tripeptides	CKK	0.0007
Tripeptides	GLK	0.0007
Tripeptides	WKK	0.0007
Dipeptides	PT	0.0007
Tripeptides	SSS	0.0006
Dipeptides	ND	0.0006
Dipeptides	PI	0.0006
Dipeptides	TR	0.0006
Dipeptides	HD	0.0006
Tripeptides	IWF	0.0006
Dipeptides	SR	0.0006
Tripeptides	STG	0.0006
Dipeptides	VC	0.0006
Tripeptides	WFQ	0.0006
Dipeptides	KW	0.0006
Tripeptides	CIC	0.0006
Tripeptides	NKP	0.0006
Dipeptides	FQ	0.0006
Tripeptides	FAV	0.0006
Dipeptides	EW	0.0006
Dipeptides	RY	0.0006
Tripeptides	HRL	0.0006
		Next Page

Group	Feature	Importance
Tripeptides	RRI	0.0006
Dipeptides	LQ	0.0006
Tripeptides	LTT	0.0006
Dipeptides	HP	0.0006
Tripeptides	GKK	0.0006
Dipeptides	GK	0.0006
Dipeptides	ST	0.0006
Dipeptides	EP	0.0006
Dipeptides	EH	0.0006
Dipeptides	LK	0.0006
Dipeptides	DD	0.0006
Dipeptides	YK	0.0006
Tripeptides	IGC	0.0006
Tripeptides	RNC	0.0006
Dipeptides	CV	0.0006
Tripeptides	NRR	0.0005
Tripeptides	PKK	0.0005
Dipeptides	GQ	0.0005
Dipeptides	VM	0.0005
Dipeptides	NG	0.0005
Dipeptides	GA	0.0005
Dipeptides	GT	0.0005
Tripeptides	PAR	0.0005
Dipeptides	QF	0.0005
Dipeptides	CR	0.0005
Dipeptides	HG	0.0005
Tripeptides	RVR	0.0005
Dipeptides	QD	0.0005
Tripeptides	RGD	0.0005
Dipeptides	VI	0.0005
Dipeptides	VL	0.0005
Tripeptides	IKI	0.0005
Dipeptides	TW	0.0005
Dipeptides	ER	0.0005
Tripeptides	RYS	0.0005
Dipeptides	TH	0.0005
Dipeptides	WI	0.0005
Tripeptides	PRR	0.0005
Dipeptides	PA	0.0005
Dipeptides	VK	0.0005
Tripeptides	RYG	0.0005
Tripeptides	ALK	0.0004
		Next Page

Group	Feature	Importance
Dipeptides	WY	0.0004
Tripeptides	PPP	0.0004
Dipeptides	PK	0.0004
Tripeptides	DKG	0.0004
Tripeptides	SNH	0.0004
Dipeptides	TF	0.0004
Dipeptides	DQ	0.0004
Dipeptides	CW	0.0004
Dipeptides	FR	0.0004
Dipeptides	RI	0.0004
Tripeptides	ACR	0.0004
Dipeptides	QA	0.0004
Dipeptides	HV	0.0004
Dipeptides	EN	0.0004
Tripeptides	AKS	0.0004
Dipeptides	AC	0.0004
Dipeptides	EF	0.0004
Tripeptides	PVL	0.0004
Tripeptides	PTT	0.0004
Tripeptides	PSS	0.0004
Dipeptides	EQ	0.0004
Tripeptides	RRC	0.0004
Dipeptides	CP	0.0004
Tripeptides	LWM	0.0004
Dipeptides	AE	0.0004
Dipeptides	VF	0.0003
Dipeptides	SI	0.0003
Dipeptides	GE	0.0003
Dipeptides	SA	0.0003
Dipeptides	EK	0.0003
Dipeptides	VA	0.0003
Tripeptides	RWY	0.0003
Tripeptides	ASR	0.0003
Dipeptides	DY	0.0003
Dipeptides	DT	0.0003
Tripeptides	SRP	0.0003
Dipeptides	HL	0.0003
Dipeptides	KK	0.0003
Dipeptides	EL	0.0003
Tripeptides	PIK	0.0003
Tripeptides	LAH	0.0003
Tripeptides	RHH	0.0003
		Next Page

Group	Feature	Importance
Dipeptides	EM	0.0003
Tripeptides	LRR	0.0003
Dipeptides	IS	0.0003
Tripeptides	PRP	0.0003
Tripeptides	WRW	0.0003
Tripeptides	KKP	0.0003
Dipeptides	FM	0.0003
Dipeptides	MD	0.0003
Dipeptides	TY	0.0003
Dipeptides	GI	0.0003
Tripeptides	HLA	0.0003
Dipeptides	TE	0.0003
Dipeptides	DC	0.0003
Tripeptides	FRF	0.0003
Tripeptides	RGR	0.0002
Dipeptides	DA	0.0002
Dipeptides	YR	0.0002
Tripeptides	GRK	0.0002
Tripeptides	WSQ	0.0002
Tripeptides	LLK	0.0002
Dipeptides	ET	0.0002
Tripeptides	AAW	0.0002
Tripeptides	CRS	0.0002
Tripeptides	WKM	0.0002
Dipeptides	MA	0.0002
Tripeptides	RWK	0.0002
Tripeptides	KKA	0.0002
Dipeptides	QK	0.0002
Tripeptides	RQI	0.0002
Dipeptides	PV	0.0002
Dipeptides	EY	0.0002
Dipeptides	YF	0.0002
Tripeptides	PWD	0.0002
Dipeptides	YD	0.0002
Tripeptides	FQN	0.0002
Tripeptides	MKI	0.0002
Tripeptides	SRA	0.0002
Tripeptides	ARA	0.0002
Tripeptides	PTL	0.0002
Tripeptides	RAA	0.0002
Tripeptides	IFI	0.0002
Tripeptides	WKC	0.0002
		Next Page

Group	Feature	Importance
Dipeptides	EC	0.0002
Tripeptides	GGR	0.0002
Tripeptides	SWR	0.0002
Dipeptides	GY	0.0002
Tripeptides	RSL	0.0002
Tripeptides	LKR	0.0002
Dipeptides	AW	0.0002
Tripeptides	RCK	0.0002
Tripeptides	GRQ	0.0002
Dipeptides	SY	0.0002
Dipeptides	AI	0.0002
Tripeptides	TPI	0.0002
Tripeptides	LRK	0.0002
Tripeptides	LLH	0.0002
Tripeptides	CRW	0.0002
Tripeptides	KKG	0.0002
Tripeptides	TRR	0.0002
Tripeptides	FRR	0.0002
Tripeptides	KAA	0.0002
Tripeptides	FST	0.0002
Dipeptides	LT	0.0002
Tripeptides	PPR	0.0002
Dipeptides	EI	0.0001
Dipeptides	TA	0.0001
Dipeptides	PY	0.0001
Tripeptides	GWT	0.0001
Tripeptides	RLR	0.0001
Dipeptides	QT	0.0001
Tripeptides	RRM	0.0001
Tripeptides	PWK	0.0001
Tripeptides	HKK	0.0001
Tripeptides	RPR	0.0001
Tripeptides	KIW	0.0001
Tripeptides	IFR	0.0001
Tripeptides	NKR	0.0001
Dipeptides	QI	0.0001
Tripeptides	TTR	0.0001
Tripeptides	RRE	0.0001
Dipeptides	WW	0.0001
Dipeptides	LI	0.0001
Tripeptides	KKT	0.0001
Tripeptides	IRR	0.0001
		Next Page

Group	Feature	Importance
Dipeptides	DM	0.0001
Tripeptides	RKR	0.0001
Tripeptides	RRS	0.0001
Tripeptides	FFN	0.0001
Dipeptides	YC	0.0001
Dipeptides	NW	0.0001
Tripeptides	KTR	0.0001
Tripeptides	PPQ	0.0001
Tripeptides	HRH	0.0001
Tripeptides	RIR	0.0001
Tripeptides	RIK	0.0001
Tripeptides	RGC	0.0001
Tripeptides	HSK	0.0001
Tripeptides	LWK	0.0001
Tripeptides	IPN	0.0001
Dipeptides	MY	0.0001
Tripeptides	VPT	0.0001
Dipeptides	MC	0.0001
Tripeptides	RFS	0.0001
Tripeptides	RRH	0.0001
Dipeptides	WR	0.0001
Tripeptides	RPK	0.0001
Tripeptides	HLR	0.0001
Dipeptides	IA	0.0001
Dipeptides	DK	0.0001
Tripeptides	KPT	0.0001
Tripeptides	KCF	0.0001
Tripeptides	PVM	0.0001
Tripeptides	RRG	0.0001
Tripeptides	RRW	0.0001
Dipeptides	CK	0.0001
Tripeptides	KWK	0.0001
Dipeptides	DW	0.0001

The t-distributed Stochastic Neighbor Embedding (t-SNE) visualization, a dimensionality reduction strategy used to visualize complex, high-dimensional data, showed a significant separation between CPPs and non-CPPs, as presented in Figure S8. All the TOP20 descriptors were used to plot this t-SNE visualization. Their combination seems to effectively clusters CPPs and non-CPPs, demonstrating their discriminative power. Thus, combining the descriptors discussed above and the small contribuitions of the others 500 features with the ERT algorithm allowed PERSEUcpp to achieve high quality results and outperform the state-of-the-art competitors.

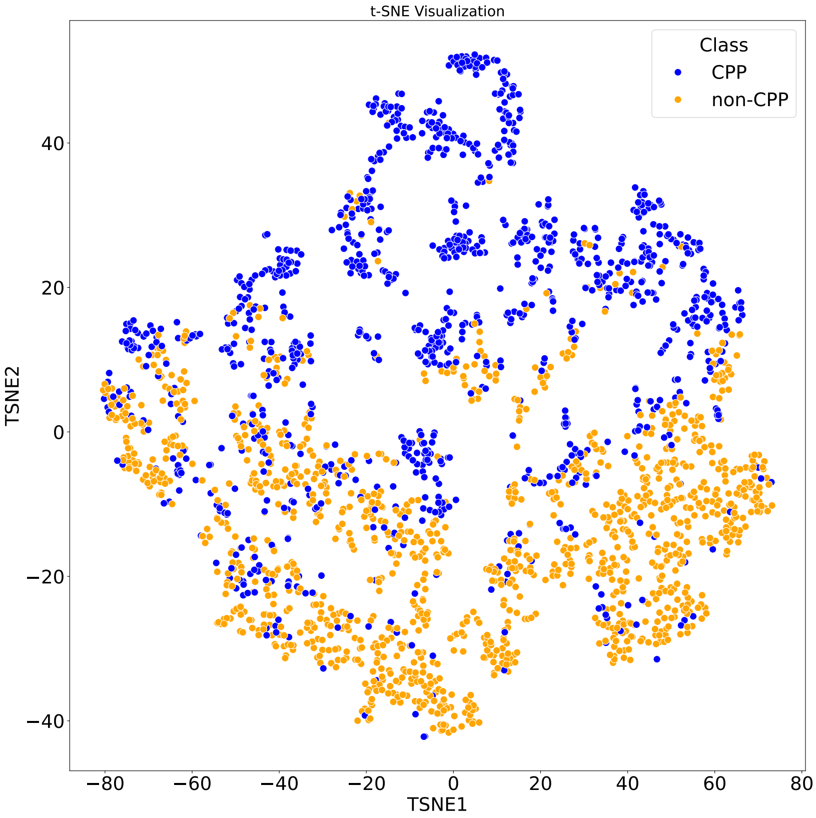

Figure S7: **t-SNE visualization of CPPs and non-CPPs**. Each point represents a peptide described by a set of calculated descriptors. Points are colored based on their classification: blue for CPPs and orange for non-CPPs. The distinct separation between the blue and orange clusters suggests that the selected features effectively differentiate between CPPs and non-CPPs.

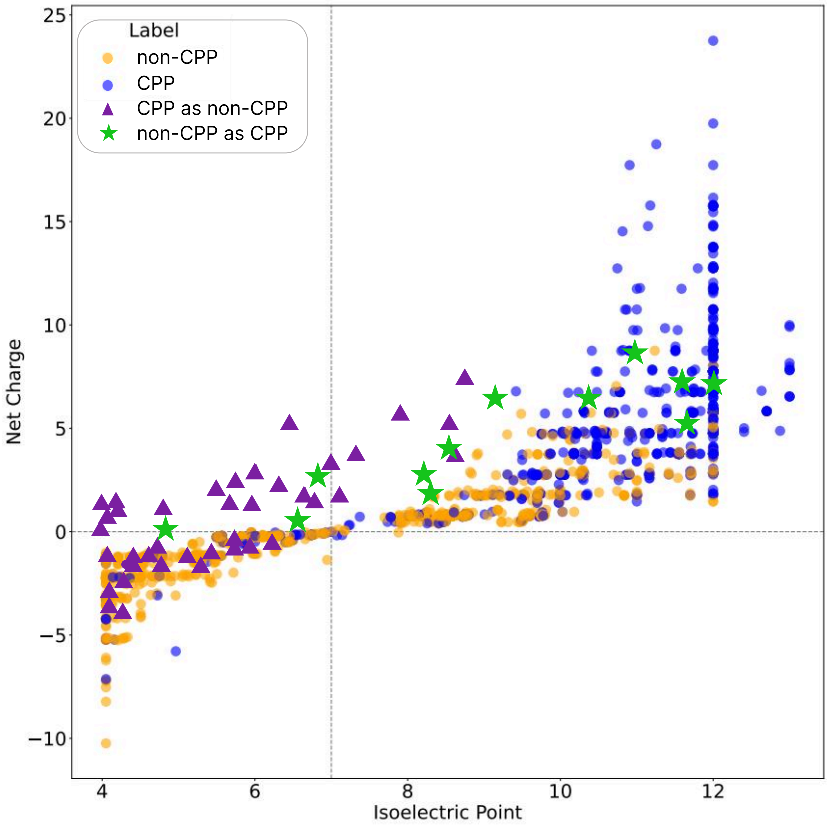

Figure S8: **Scatter plot of CPPs and non-CPPs based on isoelectric point and net charge on training dataset**. This figure is the Figure 8 presented in the Feature Importance section of the main paper; however, in addition to the points used in the training to distinguish CPPs from non-CPPs, we have also included the locations of the points that the model misclassified.

3.3 Wilcoxon signed-rank test statistic

To assess whether the observed performance differences between PerseuCPP and a leading competing method (SiameseCPP) were statistically significant, we applied the Wilcoxon signed-rank test. This non-parametric test is suitable for comparing paired measurements across matched datasets when the sample size is small or the data distribution cannot be assumed to be normal. Given the paired nature of the performance metrics (MCC, AUC, ACC, SP, and SN) across three independent test datasets, the Wilcoxon test offers a robust framework for assessing whether one model consistently outperforms the other.

The Wilcoxon signed-rank test statistic W is computed as:

$$W = \sum_{i=1}^{n} R_i \cdot \text{sgn}(x_i - y_i)$$

where R_i is the rank of the absolute difference $|x_i - y_i|$, and sgn() is the sign function that returns -1, 0, or 1 depending on the direction of the difference.

We performed the analysis using the `wilcoxon()` function from the `SciPy` library in Python. The results indicated no statistically significant differences (p-value > 0.05) across the evaluated metrics, which supports that PERSEUcpp performs competitively across multiple independent datasets. These results are summarized in Table S13.

Table S13: Comparison of PerseuCPP and SiameseCPP across independent datasets with Wilcoxon test p-values.

**Dataset: MLCPP2.0**	MCC	ACC	SN	SP	AUC
SiameseCPP	0.652	0.959	0.624	0.983	0.980
**PerseuCPP**	**0.854**	**0.989**	**0.860**	**0.994**	**0.984**
**Dataset: CPP924**	MCC	ACC	SN	SP	AUC
SiameseCPP	0.923	0.961	0.959	0.964	–
**PerseuCPP**	**0.940**	**0.970**	**0.961**	**0.978**	**0.975**
**Dataset: CPP1708**	MCC	ACC	SN	F1	AUC
GraphCPP	0.5787	0.795	0.731	0.752	0.8459
**PerseuCPP**	**0.646**	**0.811**	**0.744**	0.607	**0.865**

Bold values indicate better performance for a given metric. The Wilcoxon signed-rank test across all datasets yielded p-values of: MCC = 0.2500, AUC = 0.5000, ACC = 0.2500, SP = 1.0000, SN = 0.2500.

3.4 Case Study

To further illustrate the applicability of PerseuCPP in real-world scenarios, we conducted a case study using recently patented peptides (2023–2025) [12, 13, 14] that were experimentally validated or proposed as cell-penetrating peptides (CPPs). The objective was to evaluate how our model behaves when faced with peptides that were only recently identified, thereby assessing its predictive ability beyond benchmark datasets. Table S14 summarizes the selected CPPs, indicating the corresponding patent identifier and year of publication, the peptide sequences reported and a sequence id.

In addition to these sequences, we also performed a large-scale screening of proteins from bell pepper, sunflower, and eggplant. As a result, three novel CPPs were identified, alongside one negative control peptide. These sequences were synthesized and experimentally tested, confirming the predictions made by PerseuCPP. While the corresponding manuscript is currently under preparation, the results have already been reported in the PhD thesis [11]. Table S15 presents the peptides discovered in this screening.

Focusing on isoeletric point, charge and atomic descriptors in this independent set (7 patented peptides and 4 synthesized in-house), PerseuCPP correctly classified the 10 CPPs and the single negative control, see Table S16. CPPs showed high *isoelectric points* (median $pI = 12.44$) and *net charges* (median ≈ 5.98), consistent with the cationic profile highlighted in Figure 5 the in main paper. The experimentally validated *negative* control (EAIIQEYKEEDE), Table S16 seq-ID R3, displayed an extremely low pI ($pI = 0.00$) and the smallest cationic charge of the set (≈ 1.14), coherently reducing electrostatic attraction to anionic membrane components.

Atomic descriptors supported the same separation: CPPs concentrated around higher *nitrogen* and lower *oxygen* counts (medians $N = 2$, $O = 1$), whereas the negative control inverted this relation ($N = 1$, $O = 2$), in line with the enrichment of arginine/lysine side chains (nitrogen-rich) in CPPs and acidic residues (oxygen-rich) in non-CPPs (see Figure 5 and Figure 9 in the main paper). Hydrogen and carbon counts for CPPs centered at $H = 7$ and $C = 4$; *sulfur* showed no variability in this set and thus no discriminative effect.

Overall, the *Isoelectric Point* (pI) and *Net Charge* emerge as the primary determinants of CPP behavior, while the nitrogen–oxygen balance (N vs. O) provides complementary, sequence-level evidence. This pattern is reproduced on newly reported peptides, mirroring the decision logic learned from the training corpus. Notably, the analyses reported here were conducted with the full PerseuCPP model comprising 522 descriptors; for clarity, we discuss only a subset among the top–20 features by importance. Taken together, these results reinforce the capacity of PerseuCPP to generalize to novel sequences and to remain aligned with ongoing advances in the field.

Table S14: Patented CPP sequences (2023–2025) used for out-of-sample evaluation of PerseuCPP

Patent	Year	Peptides	Seq-ID
US20230173084A1 [12]	2023	GRKKRRQRRR	US-1
US20230173084A1 [12]	2023	GRKKRRQRRRPPQ	US-2
US20230173084A1 [12]	2023	RQIKIWFQNRRMKWKK	US-3
US11905338B2 [13]	2024	LLWRLWRLLWRLWRLL	US-4
US11905338B2 [13]	2024	LLRLLRWWRLLRLL	US-5
US11905338B2 [13]	2024	LLRLLRLLRWWRLL	US-6
WO2025038950A1 [14]	2025	RKKRRQRRR	WO-1

Each row reports the patent identifier, publication year, peptide sequence, and the internal identifier (Seq-ID) used throughout the manuscript.

Table S15: Plant-derived peptides used for out-of-sample evaluation of PerseuCPP.

Peptide Sequence	Source	Seq-ID
LLRLHGRYRLWWWWWWWY	Bell pepper	R1
WYKRRQKRRNRR	Sunflower	R2
EAIIQEYKEEDE	Sunflower	R3
RHRRNRKRPYRN	Eggplant	R4

Peptide sequences identified by large-scale screening of bell pepper, sunflower, and eggplant proteins, with their source organism and the internal identifier (Seq-ID) used throughout the manuscript. Three sequences were predicted and validated as CPPs, and one peptide (R3) is the experimentally validated negative control.

Table S16: Physicochemical and atomic descriptors for patented and plant-derived peptides.

Seq-ID	iP	NC	OAtm	NAtm	HAtm	CAtms	SAtm	Classification
US-1	8.88	4.98	1	1	5	3	0	1
US-2	11.54	6.48	1	2	7	4	0	1
US-3	14.21	7.48	1	2	8	5	0	1
US-4	14.21	5.98	1	1	8	5	0	1
US-5	12.43	5.23	1	1	7	4	0	1
US-6	12.43	5.23	1	1	7	4	0	1
WO-1	7.99	4.49	0	1	5	2	0	1
R1	13.78	6.21	1	2	8	7	0	1
R2	10.65	5.98	1	2	6	4	0	1
R3	0.00	1.13	2	1	6	4	0	0
R4	10.65	5.64	1	2	6	3	0	1

Abbreviations: iP = isoelectric point; NC = net charge; OAtm = oxygen atoms; NAtm = nitrogen atoms; HAtm = hydrogen atoms; CAtms = carbon atoms; SAtm = sulfur atoms. Seq-ID matches identifiers in Tables S14 and S15. PerseuCPP Classification: 1 = CPP, 0 = non-CPP.

4 CPP Efficiency Classifier

4.1 Feature importance

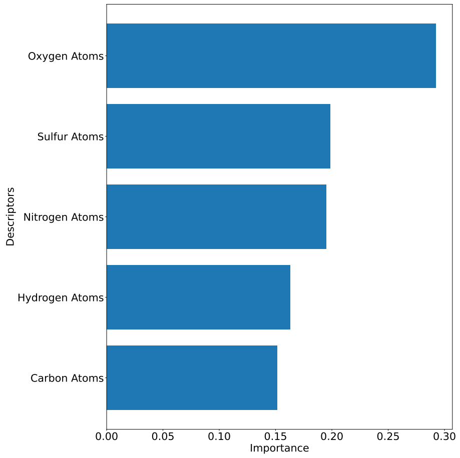

Figure S9: Top features in importance order of each atomic feature: oxygen, sulfur, nitrogen, hydrogen and carbon atoms.

4.2 PERSEUcpp compared with state-of-the-art method MLCPP2.0

Table S17: Comparison results of the proposed PERSEUcpp. ALL is all normalized features, TRIPEP is the tripeptides group, DIPEP is dipeptides group, CKSAAGP represents the Composition of k-spaced Amino Acid Group Pairs, PC is the physicochemical group and ATM is the atomic composition group of features

Descriptors	MCC	ACC	SN	SP	AUC
ALL Features	0.273	0.451	0.533	0.476	0.501
TRIPEP	- 0.092	0.451	0.456	0.437	0.465
DIPEP	0.314	0.645	0.608	0.750	0.692
CKSAAGP	- 0.052	0.580	0.695	0.250	0.565
PC + ATM + CKSA	0.145	0.645	0.700	0.423	0.631
ATM + CKSA	-0.032	0.564	0.652	0.312	0.559
ATM + DIP	-0.032	0.564	0.652	0.312	0.559
ATM + TRIP	-0.096	0.419	0.391	0.500	0.559
PC	0.314	0.645	0.608	0.750	0.692
ATM	0.356	0.726	0.761	0.625	0.690

Table S18: Comparison results of the proposed PERSEUcpp with TOP-N features of each descriptors group

Descriptors	MCC	ACC	SN	SP	AUC
TOP10	0.128	0.548	0.521	0.625	0.522
TOP50	-0.151	0.403	0.391	0.437	0.493
TOP100	-0.054	0.483	0.500	0.437	0.497
TOP150	-0.112	0.435	0.434	0.437	0.493
TOP200	-0.019	0.483	0.478	0.500	0.504
TOP250	0.019	0.516	0.521	0.500	0.513
TOP300	0.003	0.500	0.478	0.562	0.539

References

[1] Gautam, A., Chaudhary, et al. In silico approaches for designing highly effective cell penetrating peptides. *J Transl Med*, 11, 74 (2013).

[2] Holton, T.A.; Pollastri, G.; et al. CPPpred: prediction of cell-penetrating peptides. *Bioinformatics*, 2013; 29(23):3094-3096.

[3] de Oliveira, E.C.L.; Santana, et al. Predicting cell-penetrating peptides using machine learning algorithms and navigating in their chemical space. *Sci Rep*, 11:7628 (2021).

[4] Wei, L.; Tang, J.; et al. SkipCPP-Pred: an improved and promising sequence-based predictor for predicting cell-penetrating peptides. *BMC Genomics*, 18, 742 (2017).

[5] Manavalan, B.; Patra, M. MLCPP 2.0: An Updated Cell-penetrating Peptides and Their Uptake Efficiency Predictor. *J Mol Bio*, 434(11) (2022).

[6] Lundberg, S.M.; Lee, S.-I. A unified approach to interpreting model predictions. *NIPS'17*, 4768–4777 (2017).

[7] Zhang, et al. SiameseCPP: Prediction of cell-penetrating peptides using Siamese networks. *Briefings in Bioinformatics*, 23, bbac242 (2022).

[8] Kexin Shi, Yuanpeng Xiong, Yu Wang, et al. PractiCPP: a deep learning approach tailored for extremely imbalanced datasets in cell-penetrating peptide prediction. *Bioinformatics*, 40, 2, btae058 (2024).

[9] H. L. Morgan. The Generation of a Unique Machine Description for Chemical Structures-A Technique Developed at Chemical Abstracts Service. *Journal of Chemical Documentation*, 5, 2, 107-113 (1965).

[10] Imre, A., Balogh, B., Mándity, I. GraphCPP: The new state-of-the-art method for cell-penetrating peptide prediction via graph neural networks. British Journal of Pharmacology, 182(3), 495–509.

[11] Loch, R. M. B. PeptiDuo: desenvolvimento e validação experimental de um preditor computacional de peptídeos com dupla função como penetradores celulares e antimicrobianos. Tese de Doutorado, Universidade Federal de Viçosa, Programa de Pós-Graduação em Bioquímica e Biotecnologia, 2024.

[12] Tavanta Therapeutics Hungary Inc. Peptide-based synthetic chloride ion transporters. United States Patent Application, US20230173084A1, 2023. Available at: `https://patents.google.com/patent/US20230173084A1`

[13] CNRS; Université de Montpellier. Peptides for use as cell-penetrating peptides. United States Patent, US11905338B2, 2024. Available at: `https://patents.google.com/patent/US11905338B2`

[14] Knuth, M. W., Cameron, P. S., Cantor, A. J., Gowen, B. G. Cell-penetrating peptide compositions and uses thereof. World Intellectual Property Organization, WO2025038950A1, Spotlight Therapeutics, 2025. Available at: https://patents.google.com/patent/WO2025038950A1
